# Projected Impact of Salt Restriction on Prevention of Cardiovascular Disease in China: A Modeling Study

**DOI:** 10.1371/journal.pone.0146820

**Published:** 2016-02-03

**Authors:** Miao Wang, Andrew E. Moran, Jing Liu, Pamela G. Coxson, Joanne Penko, Lee Goldman, Kirsten Bibbins-Domingo, Dong Zhao

**Affiliations:** 1 Department of Epidemiology, Beijing Anzhen Hospital, Capital Medical University, Beijing Institute of Heart, Lung and Blood Vessel Diseases, Beijing, China; 2 Division of General Medicine, Department of Medicine, Columbia University, New York, New York, United States of America; 3 Division of General Internal Medicine, Department of Medicine, University of California at San Francisco, San Francisco, United States of America; 4 Columbia University College of Physicians and Surgeons, New York, New York, United States of America; Huazhong University of Science and Technology, CHINA

## Abstract

**Objectives:**

To estimate the effects of achieving China’s national goals for dietary salt (NaCl) reduction or implementing culturally-tailored dietary salt restriction strategies on cardiovascular disease (CVD) prevention.

**Methods:**

The CVD Policy Model was used to project blood pressure lowering and subsequent downstream prevented CVD that could be achieved by population-wide salt restriction in China. Outcomes were annual CVD events prevented, relative reductions in rates of CVD incidence and mortality, quality-adjusted life-years (QALYs) gained, and CVD treatment costs saved.

**Results:**

Reducing mean dietary salt intake to 9.0 g/day gradually over 10 years could prevent approximately 197 000 incident annual CVD events [95% uncertainty interval (UI): 173 000–219 000], reduce annual CVD mortality by approximately 2.5% (2.2–2.8%), gain 303 000 annual QALYs (278 000–329 000), and save approximately 1.4 billion international dollars (Int$) in annual CVD costs (Int$; 1.2–1.6 billion). Reducing mean salt intake to 6.0 g/day could approximately double these benefits. Implementing cooking salt-restriction spoons could prevent 183 000 fewer incident CVD cases (153 000–215 000) and avoid Int$1.4 billion in CVD treatment costs annually (1.2–1.7 billion). Implementing a cooking salt substitute strategy could lead to approximately three times the health benefits of the salt-restriction spoon program. More than three-quarters of benefits from any dietary salt reduction strategy would be realized in hypertensive adults.

**Conclusion:**

China could derive substantial health gains from implementation of population-wide dietary salt reduction policies. Most health benefits from any dietary salt reduction program would be realized in adults with hypertension.

## Introduction

Hypertension is a leading contributor to the epidemic of cardiovascular disease (CVD) in China [1], and high salt intake is an important behavioral cause of raised blood pressure (BP) and hypertension [2, 3]. Currently, China’s mean daily salt (NaCl) consumption is more than 12 g/day, higher than in most other countries [4, 5]. Therefore, reducing daily salt consumption has been proposed as part of China’s national strategy for non-communicable disease (NCD) prevention [6, 7].

Salt restriction goals have been set for China’s population. The objective of the national government’s Work Plan of Chronic Non-Communicable Disease Prevention was to decrease daily mean salt intake level below 9.0g during China's 12th Five-Year Plan. The goal of the China Salt Education Initiative was to reduce daily intake by a mean of 3.0g to 5.0g during 2010–2020. Potential downstream CVD health benefits of reaching national dietary salt reduction goals have not been estimated. This study integrated data from Chinese data sources into a population-scale simulation model and projected potential health benefits and health care cost savings from prevented CVD if national salt reduction goals are achieved among 35–94 years old adults in China as planned.

Salt-restriction strategies proposed for Western countries have focused on reducing salt in packaged and processed foods [8, 9]. However, these strategies are not suitable for China and other countries with similar dietary cultures, where most dietary sodium is salt added in home cooking [5, 10]. Studies in Chinese participants have provided evidence for two China-specific cooking salt reduction interventions: salt-restriction spoons and salt substitutes [11–13]. We compared the potential benefits of implementing these two dietary salt reduction interventions in all adults and only in adults diagnosed with hypertension.

## Methods

### Structure of the CVD Policy Model

**Fig 1:**
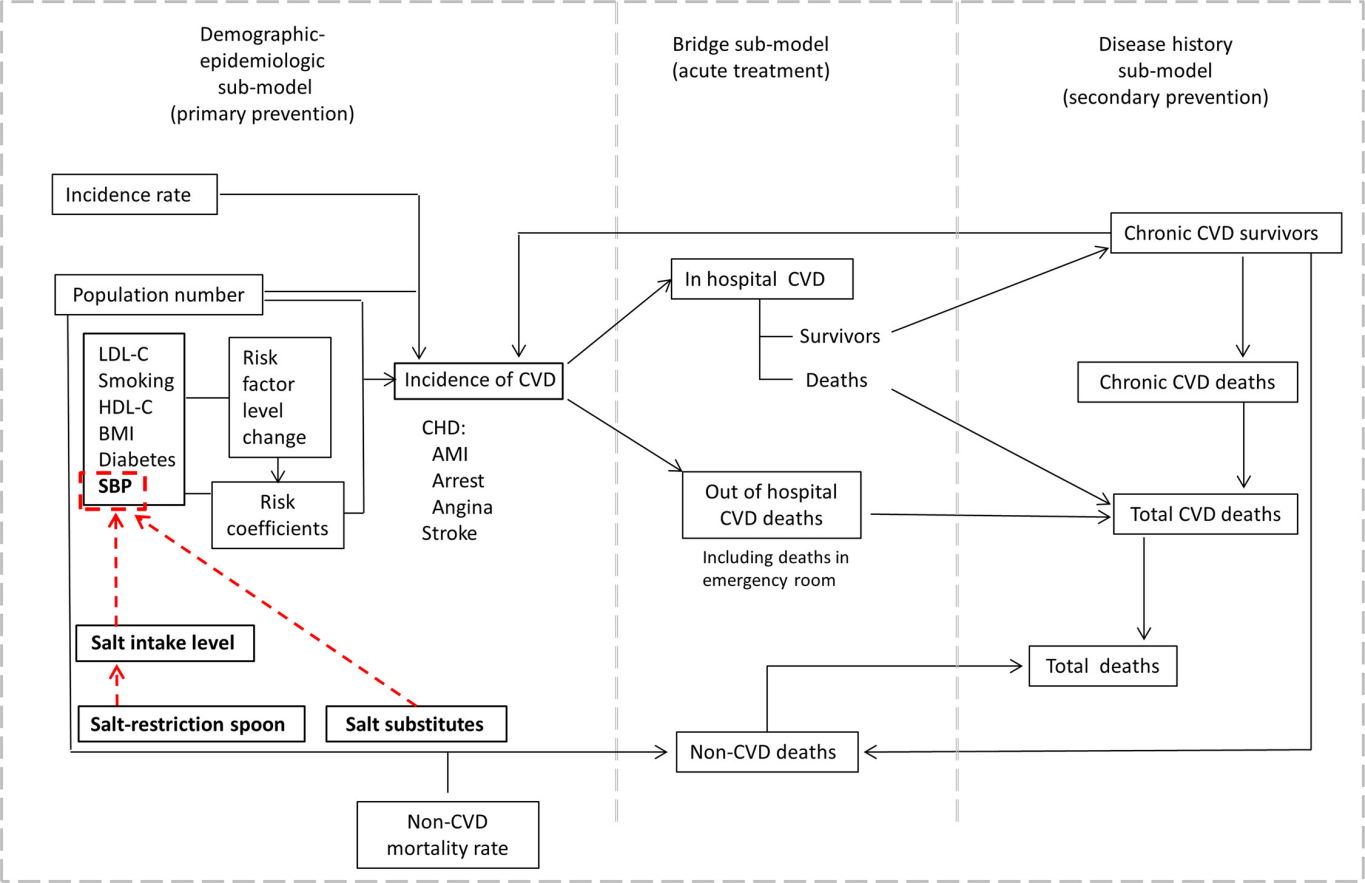
Conceptual diagram of the effect of salt reduction on the CVD prevention. AMI, acute myocardial infarction; BMI, body mass index; CHD, coronary heart disease; CVD, cardiovascular disease; HDL-C, high-density lipoprotein cholesterol; LDL-C, low density lipoprotein cholesterol; SBP, systolic blood pressure.

The potential effect of a reduction in dietary salt on CVD in China was projected by the CVD Policy Model-China, a state-transition computer simulation of coronary heart disease and stroke in China (Fig 1); model structure, default input assumptions, and validation are detailed in S1 File [14]. China-specific model inputs included population numbers, risk factor means and joint distributions, and baseline (before intervention) CVD incidence, case fatality, and mortality. Model simulations forecast the effects of risk factor exposure changes on future burden of CVD. CVD was composed of coronary heart disease (International Classification of Diseases, 10th Revision [ICD-10] codes: I20-I25 and I46) and stroke (ICD-10 codes: I60-I69). The model was stratified by 10-year age category, sex, and region (North and South China regions) because of distinctly different dietary salt intake levels, mean systolic blood pressure (SBP), and CVD incidence by age-group, sex, and region in China. All data analyses for this study involved secondary data analyses of publicly available, de-identified data. For this reason, no Institutional Review Board protocol was required for this study.

### Base Case Data Sources

Age- and sex-specific BP and other baseline risk factor levels were estimated from the International Collaborative Study of Cardiovascular Disease in Asia and a series of meta-analyses [15–19]. Mean dietary salt intake level was estimated from the Chinese National Nutrition and Health Survey conducted by National Health and Family Planning Commission of China (Table 1). Coronary heart disease and stroke incidence rates recorded by the China Hypertension Epidemiology Follow-up Study were entered into the model which was used to estimate the incidence of CVD at baseline [20]. Coronary heart disease and stroke competing risks probability functions, which estimated annual CVD event probabilities based on age, sex and of risk factor levels, were estimated from China Multi-provincial Cohort Study data using a Cox proportional hazards analysis [21]. CVD case-fatalities were from pooled Beijing Sino-MONICA Study data [22]. Stroke and coronary heart disease incidence were calibrated to match age- and cause-specific annual death rates reported by the World Health Organization and China's Health Statistics Yearbook (S1 File).

**Table 1:** Mean SBP levels, salt intake level, and relative risks of CVD associated with SBP changes within CVD Policy Model categories.

	Male (years)					Female (years)				
	35–44	45–54	55–64	65–74	75–94	35–44	45–54	55–64	65–74	75–94
**Mean SBP within category (mmHg)**										
North										
<130	118.5	117.6	118.5	118.7	120.8	112.5	115.5	117.0	120.0	121.1
130–140	136.5	136.5	136.8	138.0	139.5	134.6	135.1	135.7	135.9	136.1
> = 140	152.5	155.5	161.3	163.4	171.1	155.0	159.4	161.9	160.6	164.4
South										
<130	116.6	117.6	119.4	118.7	118.8	110.5	113.7	116.5	117.5	119.5
130–140	136.5	137.0	137.9	137.4	139.0	135.0	135.4	135.8	135.5	135.4
> = 140	153.5	156.6	161.7	161.7	163.1	154.8	156.2	157.5	158.9	164.6
**Prevalence of hypertension (%)**										
North	19.9	32.5	53.2	62.3	67.9	16.7	34.3	53.2	67.8	69.0
South	12.5	24.0	42.1	49.8	52.4	14.4	27.9	42.6	52.9	53.8
**2010 salt intake level used in the simulation (g)** *										
North	14.0	14.5	13.9	12.5	11.7	12.2	12.7	12.1	10.9	10.2
South	12.2	12.7	12.1	10.9	10.2	10.6	10.7	10.1	9.0	8.6
**Relative risk of per 10 mmHg change of SBP on coronary heart disease risk (95%CI)** †										
Nationwide	1.015	1.015	1.015	1.015	1.015	1.012	1.012	1.012	1.012	1.012
	(1.008, 1.021)	(1.008, 1.021)	(1.008, 1.021)	(1.008, 1.021)	(1.008, 1.021)	(1.002, 1.022)	(1.002, 1.022)	(1.002, 1.022)	(1.002, 1.022)	(1.002, 1.022)
**Relative risk of per 10 mmHg change of SBP on stroke risk (95%CI)**										
Nationwide	1.029	1.030	1.031	1.033	1.034	1.024	1.025	1.026	1.027	1.028
	(1.025, 1.034)	(1.026, 1.035)	(1.027, 1.036)	(1.028, 1.037)	(1.029, 1.038)	(1.019, 1.029)	(1.020, 1.030)	(1.021, 1.031)	(1.022, 1.033)	(1.023, 1.034)

*Age-, gender- and region-specific salt intake in 2010 was estimated by subtracting 0.06g per year from the mean salt intake in 2002. The rate of salt intake change was estimated based on a report from Chinese Center for Disease Control and Prevention. 1g salt (sodium chloride) = 0.393g sodium.

†, No interaction effect was observed between systolic blood pressure level and age for coronary heart disease risk.

CI, confidence interval; CVD, cardiovascular disease; SBP, systolic blood pressure.

The population in each health state in the model was assigned a quality-adjusted life year (QALY) weight and annual health care costs. Specific QALY weights were based on the Global Burden of Disease (GBD) 2010 study estimates of condition-specific disability leading to less-than-perfect health (Table 2) [23]. Number of QALYs potentially gained due to salt intake interventions were calculated by subtracting the total QALYs after intervention from the pre-intervention total.

**Table 2:** Main assumptions for the effect of salt intervention and CVD treatment costs, the CVD Policy Model-China.

Variable	Estimate (range)	Sources
**Effect of salt reduction**		A meta-analysis of effect of dietary salt restriction on blood pressure in China [3]
Effect of 1.0 g salt reduction on SBP change (mmHg/g)		
Normotensive adults	-0.55 (-0.58, -0.52)	
Hypertensive adults	-0.94 (-1.03, -0.69)	
Effect of salt-restriction spoon use on salt change (g)		
Normotensive and hypertensive adults	-1.46 (-2.40, -0.52)	
Effect of salt substitute use on SBP (mmHg)		
Normotensive adults	-2.31 (-5.57, 0.94)	
Hypertensive adults	-4.20 (-7.00, -1.30)	
**QALYs weights**		Global Burden of Disease 2010 Study [23]
**Acute state (the first 30 days after onset)**		
Nonfatal AMI	0.9064	
Nonfatal angina	0.9520	
Nonfatal stroke	0.8644	
**Chronic state**		
Nonfatal AMI	0.9648	
Nonfatal angina	0.9064	
Nonfatal stroke	0.8835	
**Death**	0.0000	
**Costs (Int$)**		
**Hospital charges**		China’s Health Statistics Yearbook 2011 [24]
AMI	4 417	
Angina	2 208	
Stroke	2 244	
Annual outpatient*		Unpublished data from Initiative for Cardiovascular Health Research [25]
The first year of coronary heart disease	909	
After the first year of coronary heart disease	633	
The first year of stroke	555	
After the first year of stroke	357	
**Per capita total expenditure on health**	245	World Health Statistics 2013[26]

*, Costs were inflated to 2010 by using inflation rate in China published by Trading Economics.

AMI, acute myocardial infarction; CVD, cardiovascular disease; Int$, international dollars (Int$1.00 = 3.53 Chinese yuan); SBP, systolic blood pressure; QALYs, quality-adjusted life years

The cost included background health care costs, acute treatment costs, and chronic state costs of CVD (Table 2) [24–26]. These costs were estimated from the health care system payer’s perspective. All expenditures were converted into international dollars (Int$) according to the exchange rate published by the World Bank, based on purchasing power parity methods (1.00 Chinese yuan = Int$0.28, Int$1.00 = 3.53 Chinese yuan). Costs and QALYs were discounted at 3% annually. In this study, we simulated mean SBP lowering expected due to dietary salt reductions. SBP reductions were in turn applied to the SBP beta coefficients in the model’s multivariate event probability functions for coronary heart disease and stroke, which translated into reductions in the projected number of incident CVD events and deaths (Fig 1). Study outcomes included projected annual numbers of prevented CVD events and deaths, relative reduction in CVD incidence and mortality rate, QALYs gained, and CVD treatment costs saved by implementing a range of salt reduction strategies. Salt reduction strategies were evaluated in the whole population and only in the population with hypertension.

The effect of a change in dietary salt on reduction of SBP and effect of salt intervention strategies were estimated in a meta-analysis of controlled feeding studies in Chinese participants (Table 2) [3]. Intervention and control groups in the salt intervention strategy trials in the meta-analysis were compared using an intention-to-treat (as randomized) analysis. The mean effect of a change in salt intake on SBP was calculated by dividing the pooled change in SBP (in mmHg) by the pooled change in reduced NaCl (in grams).

### National Dietary Salt Reduction Goals

Potential effects of dietary salt restriction in Mainland China were simulated from 2010 to 2019 in Chinese adults aged 35 to 94 years. This time range followed the time table set by the administrators of the national programs. Three simulations projected implementation of China’s national salt restriction goals within 10 years: 1) a gradual decrease to a mean of 9.0 g/day in population mean daily salt intake [7]; 2) a gradual decrease to a mean of 7.5 g/; and 3) a gradual decrease to the goal of a mean of 6.0 g/day [6]. All dietary salt change simulations were compared with a baseline in which dietary salt consumption remained at 2010 levels.

### Culturally Tailored Dietary Salt Intervention Strategies

Two specific cooking salt reduction interventions were evaluated. The first strategy promotes use of cooking salt-restriction spoons in the population, including provision of free 2-g salt spoons, teaching cooking salt measurement, and education on the potential harmful effects of high salt intake. The effect of salt restriction spoons use on SBP was based exclusively on change in dietary salt intake (Fig 1). The second cooking salt reduction strategy promotes the use of a salt substitute in the population. A currently available salt substitute in China is composed of 65% NaCl, 25% potassium chloride, and 10% magnesium sulfate [12, 13]. Both increased potassium and magnesium intake and reduce dietary salt lower mean BP [3, 27–30]. Because the sodium, potassium, and magnesium components of the salt substitute influence BP change, we input the direct BP effects observed in trials when simulating salt substitute interventions.

### Sensitivity Analyses

One-way and probabilistic sensitivity analyses examined the effects of entering the lower and upper bounds of uncertainty ranges surrounding CVD incidence risk change due to the SBP change and mean BP change per unit change in dietary salt intake. Main dietary salt restriction effectiveness assumptions were based on intention-to-treat analysis of clinical trials and therefore incorporated incomplete adherence to trial interventions. Because intervention adherence in trials is often better than in real-world settings, we also explored the effect to salt restriction strategies if the adherence rate of salt restriction strategies decreased to 75.0% and 50.0% below trials-based adherence. For example, if adherence was 75.0% below that observed in trials, the effect of the salt restriction strategy use on salt intake would also decrease by 75.0% of the main effectiveness input. Probabilistic sensitivity analyses randomly sampled 95% confidence intervals of mean SBP reduction due to dietary salt reduction estimated in the meta-analysis (Table 2) [3], and relative risks of coronary heart disease and stroke associated with changes in SBP as estimated in China Multi-provincial Cohort Study data (Table 1) [21]. In probabilistic simulations, 1000 random draws were taken from 95% CIs of the two parameters simultaneously, assuming normal distributions. Distributions of results from 1000 probabilistic simulations were used to calculate 95% uncertainty intervals (UIs) for the main outcomes of each strategy.

## Results

Assuming no change in dietary salt intake from 2010 to 2019, the simulation model projected approximately 8.2 million new CVD cases (74.2% strokes) and 2.7 million CVD deaths (59.1% stroke deaths) in Chinese adults annually (S1 Table). Projected *status quo* crude annual coronary heart disease and stroke incidence rates were 308 and 887, respectively, per 100 000 people in Chinese adults aged 35 to 94 years. Projected coronary heart disease and stroke mortality rates were 160 and 230 per 100 000 people, respectively.

### Potential Benefits of Achieving China’s National Salt-Restriction Goals

**Fig 2:**
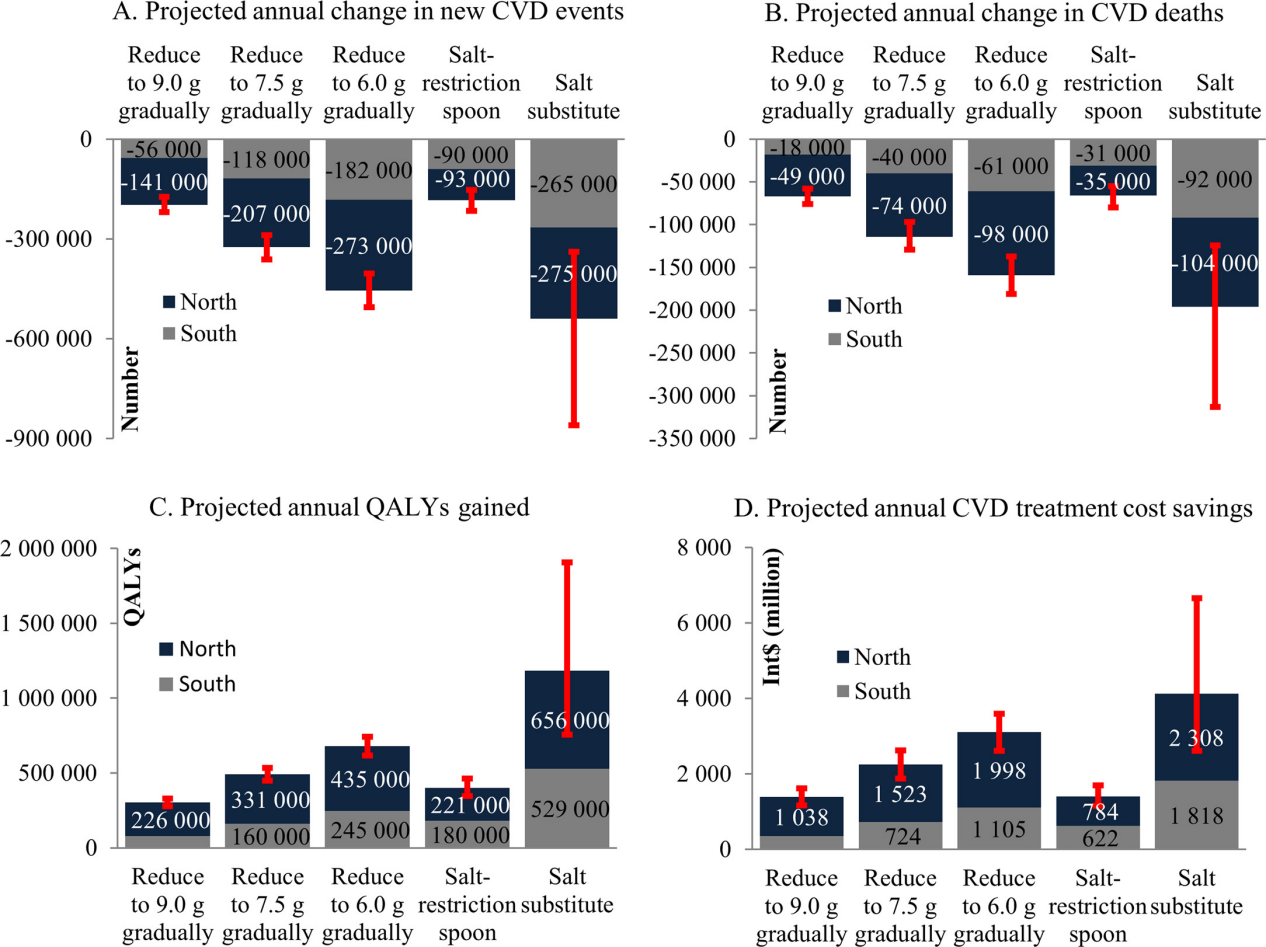
Annual benefits of CVD prevention projected for salt intake interventions by region in China. Bars represent the main simulation point estimate. I bars indicate 95% uncertainty intervals of the gained benefit among the overall population from 1 000 probabilistic simulations. CVD, cardiovascular disease; Int$, international dollars; QALY, quality-adjusted life years.

If mean daily salt intake level was reduced to 9.0 g/day from the 2010 baseline over 10 years, about 387 880 000 Chinese adults would be above the goal and eligible for such a program. Mean salt intake level would need to decrease by mean amounts varying between 1.1g to 5.5g/day because of variability in baseline salt consumption levels by sex, age-group and region (Table 1). Achieving this salt restriction goal could prevent approximately 197 000 new CVD cases (95%UI: 173 000–219 000) and 67 000 CVD deaths (58 000–76 000), and gain 303 000 QALYs (278 000–329 000) each year, as well as save approximately Int$1.4 billion in annual CVD costs (1.2–1.6) compared with no change in salt intake (Table 3 and Fig 2). Approximately 479 250 000 adults or 577 250 000 adults would be eligible for a gradual reduction of mean salt intake to goals of either 7.5 or 6.0 g/day. Achieving that goal would prevent almost 1.6 and 2.3 times the number of new cases of CVD, respectively, incremental to achieving the 9.0 g/day goal.

**Table 3:** Simulated SBP reductions and annual cardiovascular disease outcomes (coronary heart disease and stroke combined) after achieving dietary salt reduction goals in China, from 2010 to 2019, according to the CVD Policy Model-China.

	Region	Daily salt intake decreases to 9.0 g/day gradually	Daily salt intake decreases to 7.5 g/day gradually	Daily salt intake decreases to 6.0 g/day gradually
**Direct effects**				
**Target population size (N)**	**North**	188 840 000	221 760 000	256 100 000
	**South**	199 040 000	257 490 000	321 150 000
Range of mean population salt intake change (g)*	**North**	-5.5 to -1.2	-7.0 to -2.7	-8.5 to -4.2
	**South**	-3.7 to -1.1	-5.2 to -1.1	-6.7 to -2.6
**Range of mean population SBP change in normotensive adults (mmHg)** †	**North**	-3.03 (-3.19, -2.86) to -0.66 (-0.70, -0.62)	-3.85 (-4.06, -3.64) to -1.49 (-1.57, -1.4)	-4.68 (-4.93, -4.42) to -2.31 (-2.44, -2.18)
	**South**	-2.04 (-2.15, -1.92) to -0.61 (-0.64, -0.57)	-2.86 (-3.02, -2.70) to -0.61 (-0.64, -0.57)	-3.69 (-3.89, -3.48) to -1.43 (-1.51, -1.35)
**Range of mean population SBP change in hypertensive adults (mmHg)** †	**North**	-5.17 (-5.67, -3.80) to -1.13 (-1.24, -0.83)	-6.58 (-7.21, -4.83) to -2.54 (-2.78, -1.86)	-7.99 (-8.76, -5.87) to -3.95 (-4.33, -2.9)
	**South**	-3.48 (-3.81, -2.55) to -1.03 (-1.13, -0.76)	-4.89 (-5.36, -3.59) to -1.03 (-1.13, -0.76)	-6.30 (-6.90, -4.62) to -2.44 (-2.68, -1.79)
**Indirect effects**				
**Relative reduction of CVD incidence rate (%)**‡	**Overall**	2.4 (2.1, 2.7)	4.0 (3.5, 4.4)	5.6 (4.9, 6.2)
	**North**	3.0 (2.5, 3.5)	4.4 (3.7, 5.2)	5.9 (4.9, 6.8)
	**South**	1.6 (1.4, 1.8)	3.3 (2.9, 3.8)	5.1 (4.4, 5.9)
**Relative reduction of CVD mortality rate (%)**‡	**Overall**	2.5 (2.2, 2.8)	4.3 (3.6, 4.8)	5.9 (5.1, 6.8)
	**North**	3.1 (2.6, 3.7)	4.7 (3.8, 5.7)	6.3 (5.1, 7.6)
	**South**	1.6 (1.4, 1.9)	3.6 (3.0, 4.1)	5.5 (4.6, 6.5)
**Annual prevented new cases of CVD**‡	**Overall**	197 000	325 000	455 000
		(173 000, 219 000)	(288 000, 362 000)	(404 000, 505 000)
	**North**	141 000	207 000	273 000
		(118 000, 162 000)	(173 000, 241 000)	(227 000, 318 000)
	**South**	56 000	118 000	182 000
		(49 000, 64 000)	(101 000, 136 000)	(155 000, 210 000)
**Annual prevented CVD deaths**‡	**Overall**	67 000	114 000	159 000
		(58 000, 76 000)	(97 000, 129 000)	(137 000, 181 000)
	**North**	49 000	74 000	98 000
		(41 000, 58 000)	(60 000, 89 000)	(79 000, 119 000)
	**South**	18 000	40 000	61 000
		(16 000, 21 000)	(33 000, 46 000)	(51 000, 72 000)
**Annual QALYs gained**‡	**Overall**	303 000	491 000	680 000
		(278 000, 329 000)	(448 000, 535 000)	(617 000, 743 000)
	**North**	226 000	331 000	435 000
		(202 000, 250 000)	(292 000, 373 000)	(382 000, 491 000)
	**South**	77 000	160 000	245 000
		(70 000, 85 000)	(142 000, 179 000)	(217 000, 275 000)
**Annual CVD health care cost savings (millions of Int$)**‡	**Overall**	1 388 (1 158, 1 612)	2 247 (1 877, 2 616)	3 103 (2 612, 3 592)
	**North**	1 038 (823, 1 249)	1 523 (1 202, 1 869)	1 998 (1 576, 2 451)
	**South**	350 (294, 409)	724 (605, 858)	1 105 (922, 1 309)

All results represent incremental changes compared with a base case of no change from 2010 dietary salt consumption levels, with 95% uncertainty intervals estimated in probabilistic simulations.

* Changes in different sex and age groups varied because of the different baseline salt consumption levels in simulations.

† Changes in different sex and age groups varied because of the different baseline salt consumption levels in simulations. The results are shown as means and 95% confidence interval based on the effect size of salt intake change on SBP.

‡The results are shown as means from the main simulations and 95% uncertainty intervals from 1 000 probabilistic simulations. We did not account for intervention costs.

CVD, cardiovascular disease; Int$, international dollars (Int$1.00 = 3.53 Chinese yuan); SBP, systolic blood pressure; QALYs, quality-adjusted life years

### Benefits of Implementing Culturally Tailored Cooking Salt Restriction Interventions

If the salt-restriction spoon program could be successfully implemented in all adults and decreased the daily salt intake level by a population mean of 1.42g, daily salt intake level would need to decrease to 7.2g to 13.1g due to the different baseline salt intake levels. This change in dietary salt consumption could prevent approximately 183 000 new CVD cases (153 000–215 000; 1.9–2.6% relative reduction in CVD incidence) and approximately 66 000 CVD deaths annually (55 000–80 000; 2.1–3.0% relative reduction in CVD mortality rate). The salt spoon intervention could also gain approximately 401 000 QALYs (346 000–464 000) and save approximately Int$1.4 billion in CVD treatment costs annually (Int$ 1.2–1.7 billion, Table 4). If adherence to the salt-restriction spoon program decreased to 75.0% of adherence observed in trials, the intervention could still prevent approximately 137 000 new CVD cases and 50 000 CVD death, gain approximately 301 000 QALYs, and save approximately Int$ 1.1 billion in CVD treatment costs each year (S1 Table). If the adherence decreased to 50.0% of that observed in trials, the prevented new CVD cases and death would decrease to 92 000 and 34 000, the gained QALYs would decrease to 201 000, and the saved CVD treatment costs would decrease to Int$ 0.7 billion.

**Table 4:** Simulated SBP reduction and annual cardiovascular disease outcomes (coronary heart disease and stroke combined) after implementing dietary salt intervention strategies in China, 2010 to 2019, according to the CVD Policy Model-China.

	Region	Promoting the use of salt-restriction spoon		Promoting the use of substitute salt	
		In the whole population	In people with hypertension	In the whole population	In people with hypertension
Direct effects: mean population SBP change (mmHg)*					
**Normotensive**	**Overall**	-0.8 (-1.4, -0.3)	——	-2.31 (-5.57, 0.94)	——
**Hypertensive**	**Overall**	-1.4 (-2.5, -0.4)	-1.4 (-2.5, -0.4)	-4.2 (-7.0, -1.3)	-4.2 (-7.0, -1.3)
**Indirect effects**					
**Relative reduction of CVD incidence rate (%)****#**	**Overall**	2.2 (1.9, 2.6)	1.7 (1.4, 2.0)	6.6 (4.1, 10.5)	5.1 (3.6, 7.1)
	**North**	2.0 (1.5, 2.5)	1.5 (1.1, 2.0)	5.9 (3.1, 11.2)	4.6 (2.8, 7.6)
	**South**	2.5 (2.0, 3.1)	1.9 (1.5, 2.5)	7.5 (4.0, 13.7)	5.7 (3.6, 9.2)
**Relative reduction of CVD mortality rate (%)****#**	**Overall**	2.5 (2.1, 3.0)	1.9 (1.5, 2.4)	7.3 (4.6, 11.7)	5.7 (4.0, 8.0)
	**North**	2.2 (1.7, 2.9)	1.8 (1.3, 2.4)	6.7 (3.5, 12.5)	5.2 (3.1, 8.6)
	**South**	2.8 (2.2, 3.5)	2.1 (1.6, 2.8)	8.3 (4.5, 15.2)	6.3 (4.0, 9.9)
**Annual prevented new cases of CVD****#**	**Overall**	183 000	140 000	540 000	418 000
		(153 000, 215 000)	(115 000, 167 000)	(339 000, 860 000)	(295 000, 585 000)
	**North**	93 000	72 000	275 000	215 000
		(71 000, 117 000)	(53 000, 94 000)	(144 000, 519 000)	(129 000, 354 000)
	**South**	90 000	68 000	265 000	203 000
		(71 000, 111 000)	(52 000, 88 000)	(142 000, 486 000)	(127 000, 325 000)
**Annual prevented CVD deaths****#**	**Overall**	66 000	51 000	196 000	152 000
		(55 000, 80 000)	(41 000, 63 000)	(124 000, 313 000)	(107 000, 215 000)
	**North**	35 000	28 000	104 000	82 000
		(26 000, 46 000)	(20 000, 37 000)	(54 000, 196 000)	(49 000, 134 000)
	**South**	31 000	23 000	92 000	70 000
		(25 000, 39 000)	(18 000, 31 000)	(50 000, 169 000)	(44 000, 110 000)
**Annual QALYs gained****#**	**Overall**	401 000	292 000	1 185 000	876 000
		(346 000, 464 000)	(245 000, 349 000)	(756 000, 1 906 000)	(633 000, 1 226 000)
	**North**	221 000	162 000	656 000	487 000
		(177 000, 275 000)	(125 000, 210 000)	(347 000, 1 240 000)	(302 000, 784 000)
	**South**	180 000	130 000	529 000	389 000
		(146 000, 218 000)	(102 000, 166 000)	(288 000, 979 000)	(251 000, 615 000)
**Annual CVD health care cost savings (millions of Int$)****#**	**Overall**	1 406 (1 154, 1 694)	1 061 (853, 1 314)	4 126 (2 607, 6 654)	3 149 (2 223, 4 519)
	**North**	784 (575, 1026)	600 (426, 814)	2 308 (1 182, 4 397)	1 785 (1 057, 2 958)
	**South**	622 (487, 781)	461 (346, 602)	1 818 (978, 3 365)	1 364 (861, 2163)

All results represent incremental changes compared with a base case of no change from 2010 dietary salt consumption levels, with 95% uncertainty intervals. Since the two simulated salt restriction strategies were family-based, Chinese adults whose salt intake level were higher than the recommended amount and their family numbers may both be affected.

*, Average change in the population level. Changes in different sex and age groups were the same during simulations.

#,The results are shown as means from the main simulations and 95% uncertainty intervals from 1 000 probabilistic simulations. We did not account for intervention costs.

CVD, cardiovascular disease; Int$, international dollars (Int$1.00 = 3.53 Chinese yuan); SBP, systolic blood pressure; QALYs, quality-adjusted life years

Salt substitutes would be even more effective, potentially preventing approximately three times more incident CVD events and deaths compared with the salt-restriction spoon intervention if fully taken up by all adults. Implementing a cooking salt substitute strategy in all adults could prevent approximately 540 000 (339 000–860 000) new CVD events annually (Table 4); more than the benefit that we projected for decreasing the mean salt intake level to 6.0 g/day gradually in 10 years. However, the 95% UI around the salt substitute estimate was wide and included estimates well below the lower bound of the 95% UI of effect size for the national 6.0 g/day goal (Fig 2). If adherence decreased to 75.0%, the implementation of the salt substitutes strategy would still prevent approximately 409 000 new CVD cases and 149 000 CVD death, gain approximately 895 000 QALYs, and save approximately Int$ 3.1 billion in CVD treatment costs each year (S1 Table). If adherence rate lower to 50.0%, the prevented new CVD cases and death would decrease to 274 000 and 100 000, the gained QALYs decrease to 601 000, and the saved CVD treatment costs would decrease to Int$ 2.1 billion.

### Benefits of Two Culturally Tailored Strategies for Cooking Salt-Restriction in Adults with Hypertension

**Fig 3:**
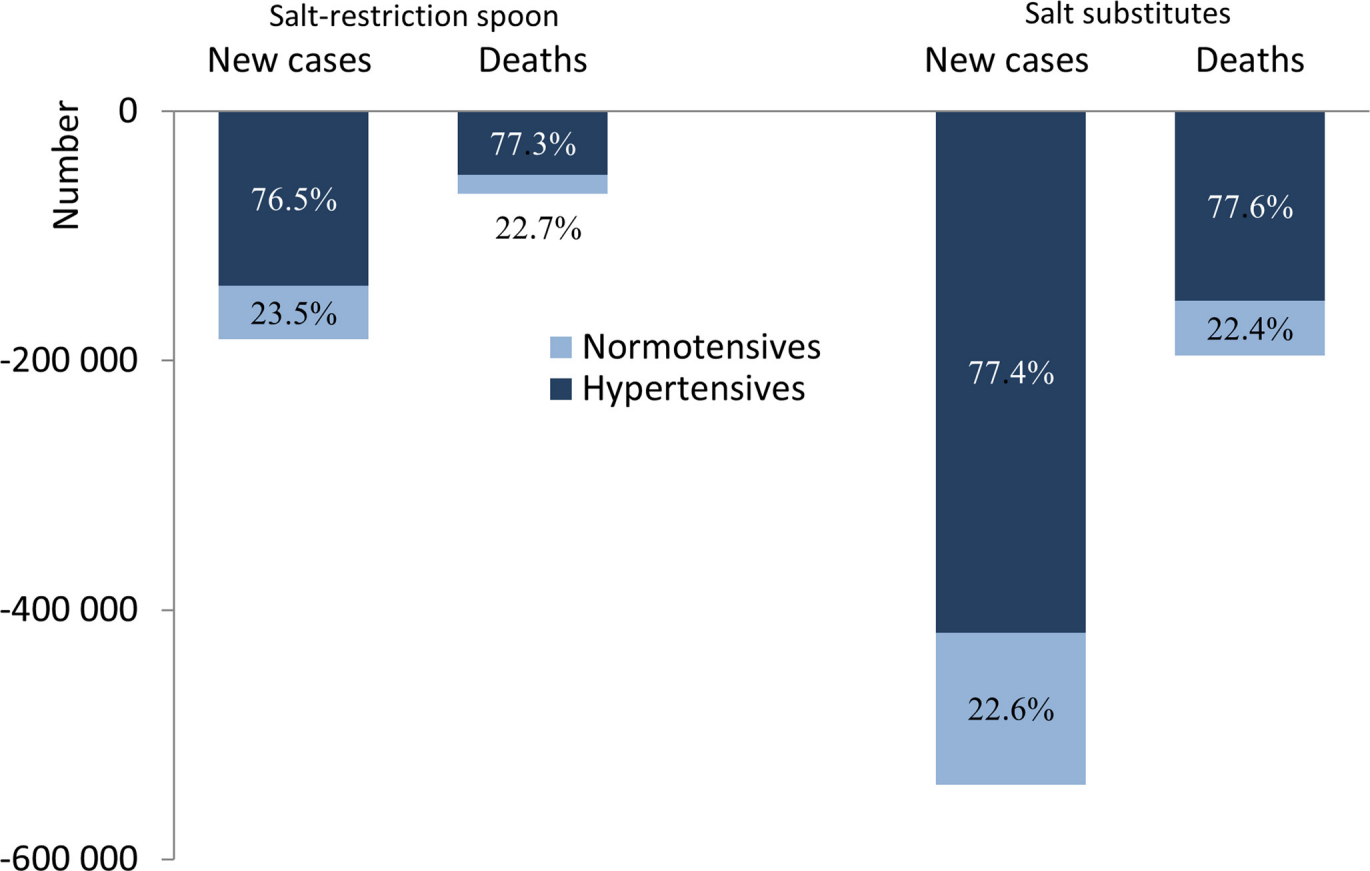
Simulated annual number of CVD events prevented by salt restriction strategies by hypertension status.

Approximately three-quarters of the CVD prevention benefits for each cooking salt-restriction strategy would be among adults with hypertension. For the salt-restriction spoons intervention, 76.5% of new cases of CVD, 77.3% of CVD deaths, 72.8% of QALYs gained, and 75.4% of costs savings would be realized in the population with hypertension (Table 4 and Fig 3). Similarly, 80% of the projected reduction in CVD incidence and mortality rates from the salt substitute strategy would be realized among people with hypertension.

### Sensitivity Analyses

The magnitude of benefits of prevention of CVD and cost savings did not substantially vary in one-way sensitivity analyses, except for the salt substitute strategy, which was affected by a wider range of uncertainty, reflecting a wide range of BP-lowering effects observed in salt substitute trials (Fig 2 and Table 4). The 95% UI for the projected change in CVD incidence rate achievable with a salt substitute intervention had a lower bound of 4.1% and an upper bound of 10.5% (Table 4).

## Discussion

This study projected the magnitude of CVD prevention that is potentially achievable if one of several population-wide dietary salt reduction strategies were to be implemented in China. Depending on the strategy, we found that dietary salt reduction could prevent between 183 000 and 540 000 new CVD cases and save between Int$1.4 and Int$ 4.1billion in CVD treatment costs annually. Adults with hypertension would realize the most health gains from adopting specific dietary salt reduction interventions, such as salt restriction spoons or salt substitutes.

Data from Chinese National Nutrition and Health Survey in 2002 shown the mean daily salt intake level was 12.0g and varied by sex, age, and region (Appendix Table 3 in S1 File) [31]. News form Chinese Center for Disease Control and Prevention which conducted the new Chinese National Nutrition and Health Survey (data was still not published) shown the mean salt intake level in urban Chinese only decreased by 0.6g from 2002 to 2012 [32]. Another large survey enrolled 16 869 participants from 9 provinces in China and shown the mean salt intake level were about 12.0g/day in 2009 [33]. Both results shown the recent mean daily salt intake level was more than twice the WHO recommended salt intake (<5.0g/day). Salt restriction still was a major health problem in China.

Using the same simulation model, we previously projected that lowering BP with antihypertensive drugs in Chinese adults with hypertension could prevent about 803 000 CVD events annually [34]. In this study, we projected that implementing a cooking salt substitute in people with hypertension could prevent approximately 540 000 CVD events annually. A dietary salt reduction strategy could complement pharmacological treatment of raised BP in adults with hypertension, and possibly extend the health benefits of a reduction in BP to those with elevated BP, but not meeting the criteria for the diagnosis of hypertension. Because the program costs of dietary salt-lowering strategies are not well defined, we did not account for programmatic costs or estimate net cost-effectiveness.

A recent study estimated that improved control of six major risk factors in all countries would reduce global NCD mortality by approximately 19%, approaching the World Health Organization goal of a 25% reduction in NCD mortality by 2025 [35]. We projected that decreasing mean salt intake levels to 9.0 g or 6.0 g gradually according to national goals could decrease CVD mortality by a mean of 2.4% or 5.6% from 2010 to 2019, and decrease NCD mortality by 1.2% or 2.7% (estimated based on data from the China’s Health Statistical Yearbook 2011; NCDs included CVD, cancer, chronic respiratory disease, and diabetes) [24].

Achieving salt-restriction goals in China and other countries will require policies adapted to the local context. North American and European studies have suggested that a reduction in salt intake could be implemented in packaged, “processed” foods, requiring some combination of voluntary cooperation by the food industry or legislation [8, 9, 36, 37]. These “supply side” strategies are likely to be less relevant for China and other countries where the primary source of dietary salt is home-cooked foods [5]. Our review identified two interventions that focused on reducing cooking salt use [3]. The salt substitute strategy was projected to have a greater effect than salt-restriction spoons, though with a wide uncertainty interval (Table 4). Salt substitute strategy appeared to achieve the same or greater benefits as achieving the 6g/day goal in 79.4% of probabilistic simulations.

In our main projections, we assumed that a larger decrease in dietary salt would lead to a greater reduction in BP and greater downstream health benefits. However, the compliance with the salt restriction strategies was a key factor that could change the benefits of CVD prevention from the salt restriction. Our result suggest that if the adherence to the salt-restriction spoon intervention decreased to 75.0% or 50.0% of adherence observed in intervention trials, prevented new CVD cases would decrease to 74.9% or 50.3% of the main projections (S1 Table). If adherence to salt substitutes decreased to 75.0% or 50.0% of adherence observed in trials, the benefits of CVD prevention from the salt substitutes strategy would decrease by about quarter or one half. The salt-restriction spoon strategy employed an educational intervention in order to influence behavior change, but ultimately depended on participants’ adherence to the intervention.

For the salt substitute strategy, the benefits of salt substitutes on BP may come from the decrease of sodium intake level and the increase of potassium intake level. Data from current meta-analysis of salt substitutes studies shown the level of urinary potassium did increase, but the level of urinary sodium seemed unchanged during intervention [3, 38]. This result suggests that lowering of BP from salt substitutes might have been due to increased potassium intake alone. This is plausible because salt substitute users may titrate the substitute dose to achieve the sodium taste they are accustomed to thereby matching their baseline sodium intake (keeping sodium intake constant) but thereby increasing potassium and magnesium intake—which could still lower BP overall due to the BP-lowering effects of potassium and magnesium.

The impact of potassium intake from salt substitutes on serum potassium level has not been well established. In our previous meta-analysis, only one study reported the effect of salt substitutes use on serum potassium level and showed no effect [3, 12]. Before large scale implementation of a salt substitute strategy, the relative risk of hyperkalemia in people receiving the intervention should be better established.

## Limitations

We selected China-specific, nationally representative data for model inputs. Simulated stroke and coronary heart disease mortality outputs matched well with two sources of cause-specific mortality from China (S1 File). However, similar to other computer simulation studies, this study was limited by reliance on multiple assumptions and data inputs from diverse studies. We assumed that dietary salt lowering, leads to BP reduction, and consequently to reduced CVD risk. The Prospective Urban Rural Epidemiology (PURE) study observed that participants were exposed to lower levels of sodium (NaCl intake levels <7.5 g/day) may increase the risk of all-cause mortality [39]. If observational data from the PURE study are extrapolated to an intervention context, reducing mean salt intake to 6.0 g/day would shift a proportion of the population starting in the lower range of salt consumption at baseline to a potentially unsafe level. The hypothetical harms from such a shift would be relatively rare in a high-salt consuming population like China’s, and harms suggested by the PURE data would have to be multiplied eight-fold or more to eclipse the benefits of prevention of CVD that we projected. Our estimate of a change in BP with salt in people with normal BP was based on studies of hypertensive and normotensive participants combined. Thus, we may have overestimated the magnitude of BP lowering achievable in normotensive adults. The estimated effect of changes in salt intake on BP in Chinese studies was mainly estimated from studies with short-term follow up studies which may not sustainable and so may not represent long-term effects. We also lacked sufficient data to establish a dose-response relationship between dietary salt consumption and BP. Reassuringly, larger international analyses found no significant differences in BP responses when short- versus long-term follow-up studies were compared, and that a linear association between salt intake and BP can be assumed [2, 40]. Salt restriction strategies in China would require initial investment in the cost of salt-restriction spoon or difference between salt substitutes, cost of education on the health risks associated with high salt intake and how to implement the intervention. Currently, no study reported costs of the two salt restriction strategies. Because of this, costs of the strategy were not accounted for in our analysis. Total health care costs would include offsets for intervention costs and any adverse effects; as a result our cost-savings estimates are likely overly optimistic.

In conclusion, implementation of dietary salt lowering in Chinese adults could be an effective population-wide approach to high BP control and prevention of CVD. Most of the benefits of dietary salt reduction would be realized in people with hypertension. Cooking salt substitutes strategy may be more effective than salt-restriction spoons strategy.

## Ethics Committee Approval

All data analyses for this study involved secondary data analyses of publicly available, de-identified data. For this reason, no ethics board approval was sought for this study.

## Supporting Information

S1 File Modeling methods and data resources. (DOCX)

S2 File Creative Commons agreement. (DOC)

S1 Table Results of one-way sensitivity analyses of the potential effects of salt restriction on CVD prevention. (DOCX)
