# Supplementary material for: Projected Impact of Salt Restriction on Prevention of Cardiovascular Disease in China: A Modeling Study
Source: PLoS One. 2016 Feb 3;11(2):e0146820. doi: 10.1371/journal.pone.0146820 (PMC4739496; doi:10.1371/journal.pone.0146820)
Supplement: S1 File — (DOCX) [file pone.0146820.s001.docx]

**Modeling methods and data resources**

1. **Description of the Cardiovascular Disease (CVD) Policy Model-China computer simulation model**
2. **Model calibration**
3. **Analysis assumptions related to dietary salt intake**

**1. Description of the Cardiovascular Disease (CVD) Policy Model-China computer simulation model**

The CVD Policy-China is a Markov (state-transition) model of CVD in the adult Chinese population [[1](#_ENREF_1), [2](#_ENREF_2)]. Means and proportions of cardiovascular disease risk factors in Chinese adults in ten-year age categories ages 35-94 years in 2000 were estimated from the International Collaborative Study of Cardiovascular Disease in Asia Study (InterASIA) [[3](#_ENREF_3)]. CVD incidence[[4](#_ENREF_4), [5](#_ENREF_5)], mortality [[6](#_ENREF_6)], and case-fatality[[4](#_ENREF_4)] estimates were derived from other Chinese studies.

For this analysis CVD was composed of coronary heart disease (International Classification of Diseases, 10th Revision [ICD-10] codes: I20-I25 and I46) and stroke (ICD-10 codes: I60-I69). In the model, population aged 35-94 years old is described in four main states: healthy (free of coronary heart disease or stroke), acute CVD (up to 30 days following symptom onset), chronic CVD states (coronary heart disease alone, stroke alone, or coronary heart disease and stroke combined), or death (Fig 1 in main text and Appendix Fig 1). People turning 35 years old in years subsequent to the base year were added with successive annual cycles and the population reaching ages greater than 94 years are censored from the model. The base case or *status quo* incidence of CVD was projected using the healthy population at risk and age- and sex-specific CVD incidence rates.

Prior to model calibration, CVD incidence and non-CVD death rates estimated from the China Hypertension Epidemiology Follow-up Study (CHEFS) were entered into the CVD Policy Model [[5](#_ENREF_5)]. A multi-stage, random clustering design was used to identify a nationally representative sample of Chinese men and women >= age 15 years beginning in 1991 living in 30 provinces of China. In 1999, investigators from each province were invited to participate in the CHEFS. Seventeen provinces participated in the follow up study. 83 533 men and 86 338 women older than age 40 at the time of the 1991 baseline examination were eligible for follow-up. Follow up disease event data were gathered from 158 666 participants or proxies by interview in 1999-2000 (follow-up rate was 93.4%). Urban and rural status was determined according to China census designations, and North and South China were divided by the Yangtze River. In follow up examinations during 1999 and 2000, participants or their proxies were tracked to a current address and in-depth interviews were conducted in order to obtain information on history of disease, hospitalizations, or deaths. For deaths, death certificates were obtained from local public health or police departments. For hospitalized CVD events or deaths, hospital records were obtained, including physical examination, laboratory findings, electrocardiograph records, brain imaging, and autopsy findings were included in the evaluation of cases. An end-point assessment committee in each province reviewed and confirmed (or rejected the hospital discharge diagnosis or cause of death on the basis of abstracted information, using prespecified criteria). A study-wide endpoint assessment committee at the Chinese Academy of Medical Sciences in Beijing, consisting of cardiologists, neurologists, and a clinical epidemiologist reviewed all diagnostic and cause of death information and determined the final diagnosis or cause of death. Two committee members independently verified diagnosis or cause of death and disagreements were adjudicated after discussion by other committee members. Cases were also classified according to two versions of the International Classification of Diseases, ICD-9 and ICD-10.

**Appendix Fig 1.**

Transition 1 = remain in CVD-free state; Transition 2 = incident CVD; Transition 3 = non-CVD death; Transitions 4 and 5 = survival or case-fatality; Transition 6 = survival with or without repeat CVD event in chronic CVD patients.

BMI, body mass index; CHD, coronary heart disease; CVD, cardiovascular disease; HDL-C, high-density lipoprotein cholesterol; LDL-C, low density lipoprotein cholesterol; MI, myocardial infarction

Risk factor levels at baseline are age-specific estimates from the InterASIA (2001-2002) and trended to the base year of 2010 based on a published risk factor trend analysis [[7-10](#_ENREF_7)]. InterASIA purposely specified distinct sampling frames for North and China, allowing estimation of risk factors for these two regions separately. Mean blood pressure was the average of the second two of three seated measurements, measured after a five minute wait by a trained observer and using a standard and calibrated mercury sphygmomanometer and appropriate cuff size. Hypertension status and hypertension awareness were established based on self-reported physician diagnosis, report of taking anti-hypertensive drugs, and measured blood pressure.

The age-trend for each risk factor (e.g. higher SBP with higher age, lower cholesterol in oldest age category) was preserved by a risk factor transition function in the model software: all base year (year 2000) risk factor levels are age- and sex-specific and taken from the nationally-representative InterASIA study (male and female and age categories 35-44, 45-54, 55-64, 65-74, 75-84 and 85-94 years). The CVD Policy Model software includes a function that ensures that the age-trend in risk factor levels is preserved as age cohorts upward and new waves of 35 year olds enter with each successive year of the model simulation. Transfers from one risk factor level to another are included to ensure that the InterASIA proportions of the population with each risk factor level are maintained. Transfers from one risk factor level to another were included to preserve the InterASIA Study proportions of the population with each risk factor level. For example, the proportion of 35-44 year old men with low low density lipoprotein (LDL) cholesterol (<100 mg/dL) is 0.482. For 45-54 year old men the proportion is 0.437. The shift toward higher LDL cholesterol levels is most likely caused by increasing LDL levels as people age. In higher age ranges, this trend reverses, so that by age 75-84, the proportion is 0.403. The change in the upper age ranges is most likely due to a more complex array of factors, including the fact that people with higher risk are more likely to die. Annual transfer rates between risk factor levels were calculated to reduce the low risk population from 0.215 to 0.133 over 10 years, without regard to the reason for the change, but taking into account the effect of the Model’s CVD incidence and non-CVD death rates. As the age composition of the population changes (specifically, in China the population shifts to a relatively larger proportion of the population in older age groups), risk factor exposures change due to a larger proportion of the population exposed to the risk factor levels characteristic of the older age categories and a smaller proportion exposed to risk factor levels characteristic of the younger population.

Annual probability of first CVD events and non-CVD deaths conditioned on demographic and risk factors were estimated by analyzing the China Multi-provincial Cohort Study (CMCS). The CMCS was a cohort study of 30 378 male and female participants aged 35-64 years and with no cardiovascular disease at baseline in 1992-1993 (more details about the CMCS provided in a manuscript by Liu et al) [[11](#_ENREF_11)]. These participants were recruited from 16 centers in 11 Chinese provinces using a multistage sampling method. Twelve centers (80.3% of participants) were in urban areas and the remainder in rural areas. Overall baseline participation rate was 82%. Baseline measurement of risk factors using a standard protocol (WHO-MONICA protocol) and blood samples were processed at a central laboratory [[12](#_ENREF_12)]. Case-finding of new CHD and stroke events and non-cardiovascular deaths was first done by face-to-face interview. Events were ascertained by 1) detailed interview of participants or family members, 2) review of hospital records. These events were later adjudicated by investigators at the Beijing Institute of Heart, Lung, and Blood Vessel Diseases. After 1996, six centers ceased follow up because of completion of that national research project, but the remaining ten centers (16 552 participants) were followed up through the end of 2002. Follow up rate was 86% for the centers followed all of 1992-2002, and 65% of the original 16 center cohorts. Multivariate Cox proportional hazard ratios for systolic blood pressure (SBP), diabetes mellitus, total cholesterol, body mass index, and active cigarette smoking were estimated from baseline measurements and ischemic and hemorrhagic events occurring over 159 400 person-years of observation in CMCS participants [[11](#_ENREF_11)]. Significant (*P* < 0.05) age*risk factor risk coefficient interactions (higher risk at higher ages) were observed for smoking in CMCS multivariate CHD models, SBP and smoking in total stroke models, and smoking and diabetes in non-cardiovascular mortality models, so these were incorporated in age-specific risk coefficients.

Main CVD Policy Model-China 28-day case fatality assumptions were estimated from pooled Beijing Sino-MONICA Study data from 1993-2004 (personal communication, Dong Zhao, MD, PhD, 2006) and the main age-specific CVD case-fatality rate assumptions were estimated from the overall rates. Annual probabilities of repeat CVD events, probability of stroke after myocardial infarction and the probability of coronary heart disease in stroke patients were based on natural history studies [[13-21](#_ENREF_13)]. Intervention simulations project a change in CVD incidence with changes made to blood pressure or any other risk factor. Acute event survivors transition to the chronic CVD state after 30 days. The model cycle is one year. All resources were listed in Appendix Table 1.

**Appendix Table 1. Parameters of the CVD Policy Model-China**

| **Model component** | **Model Outputs** | **Input parameters** | **Sources *** |
| --- | --- | --- | --- |
| Demographic-epidemiologic sub-model (population without CVD) | Number of first-ever CVD events | Population number aged 35-94 years old | The Sixth National Population Census [[22](#_ENREF_22)] |
|  |  | Incidence rate of CVD | China National Hypertension Survey Epidemiology Follow-up Study (CHEFS) [[5](#_ENREF_5)] |
|  | Change in probability of first-ever CVD events due to the risk factor level change | Population number aged 35-94 years old | The Sixth National Population Census |
|  |  | Level of risk factors | International Collaborative Study of Cardiovascular Disease in Asia (InterASIA) [[3](#_ENREF_3)] |
|  |  | Risk coefficients | Published and unpublished data from China Multi-provincial Cohort Study [[11](#_ENREF_11)] |
| Bridge  sub-model (acute CVD states) | Number of CVD survived from the acute stage  Number of CVD deaths in the acute stage  Number of hospitalized CVD | 30-day case fatality rate | Published and unpublished data from Multinational Monitoring of Trends and Determinants in Cardiovascular Disease (MONICA) study [[4](#_ENREF_4)] |
|  |  | Proportion of hospitalized CVD of all cases |  |
| Disease history  sub-model (chronic CVD states) | Number of acute coronary events with CVD history  Number of yearly chronic CVD deaths | Population aged 35-94 years old | The Sixth National Population Census |
|  |  | Prevalence of chronic CVD | InterASIA Study self-report |
|  |  | Probability of CVD events in people with CVD history | Natural history studies [[13-21](#_ENREF_13)] |

*, includes published and unpublished data. CVD, cardiovascular disease; CHD, coronary heart disease

**2. Model calibration**

**Comparison with mortality rate targets for China**

Target total mortality for the Chinese adults aged 35-84 years in 2010 were obtained from the World Health Organization (WHO) and China's Health Statistics Yearbook 2011 which was published by the National Health and Family Planning Commission (NHFPC) of China. Pre-calibration age- and sex-specific CHD and stroke mortality rates in the model were estimated from the CHEFS. The population estimates for 2010 were obtained from the Sixth National Population Census and entered into the model (Appendix Table 2).

**Appendix Table 2. Chinese population by age, sex, and region categories in 2010**

| **Age Group**  **(years)** | **North** | | **South** | | **Total** |
| --- | --- | --- | --- | --- | --- |
|  | **Male** | **Female** | **Male** | **Female** |  |
| **35-39** | 24 847 142 | 23 780 107 | 35 543 962 | 33 854 748 | 118 025 959 |
| **40-44** | 27 005 642 | 25 997 714 | 36 603 036 | 35 147 572 | 124 753 964 |
| **45-49** | 23 552 009 | 22 970 838 | 30 224 409 | 28 847 297 | 105 594 553 |
| **50-54** | 18 788 121 | 18 106 979 | 21 575 113 | 20 282 958 | 78 753 171 |
| **55-59** | 18 151 547 | 17 951 431 | 22 931 391 | 22 278 105 | 81 312 474 |
| **60-64** | 12 590 861 | 12 583 984 | 17 243 565 | 16 248 872 | 58 667 282 |
| **65-69** | 8 444 959 | 8 619 048 | 12 303 512 | 11 745 763 | 41 113 282 |
| **70-74** | 6 725 244 | 6 915 277 | 9 678 209 | 9 653 667 | 32 972 397 |
| **75-79** | 4 555 853 | 5 044 038 | 6 723 006 | 7 529 236 | 23 852 133 |
| **80-84** | 2 308 264 | 2 824 865 | 3 609 238 | 4 630 831 | 13 373 198 |
| **85-94** | 1 031 417 | 1 586 105 | 1 699 265 | 2 893 448 | 7 210 235 |
| **Total** | 148 001 059 | 146 380 386 | 198 134 706 | 193 112 497 | 685 628 648 |

In the calibration procedure, CHD and stroke parameters were calibrated separately. Starting with default incidence, case-fatality, and prevalence assumptions, parameters were calibrated to remain consistent with external source targets from 2004-2010, as well as achieve an estimate midway between WHO and China NHFPC mortality numbers in 2010, as there was no evidence that either source was of higher quality than the other. Simulated absolute numbers of CHD and stroke deaths were within less than 1% of the average of the numbers of observed deaths reported by the WHO and NHFPC in 2010 (that is, midway in-between the estimates from these two sources).

In order to evaluate the accuracy of CVD Policy Model predictions over time, China CVD mortality for ages 35-84 years were compared with WHO and NHFPC estimates for the years 2004-2010. Appendix Figs 2A-2D below demonstrate that CVD Policy Model predictions are reasonably close to NHFPCC and WHO estimates for 2007-2010, but did not predict as well for 2004-2006. CHD mortality numbers from 2007-2010 were varied by 12% or less in from the average of the observed numbers estimated by the NHFPCC and WHO males and 8% or less from the average in females. For stroke, numbers of deaths varied less than 5% from the average of NHFPCC and WHO estimates in male and less than 2% from the average in females.

WHO, World Health Organization; NHFPC, National Health and Family Planning Commission

**3. Analysis assumptions related to dietary salt intake**

**Effect of salt intake change on CVD burden**

For this study, we assumed that decreased salt intake would lead to a decrease in systolic blood pressure (SBP), and that secondly, via the SBP beta coefficients and the change in mean SBP in the CVD probability functions, decreased SBP lowers CVD incidence (Fig 1 in the main text). Considering the regional differences in population, mean dietary salt intake, and blood pressure, the CVD Policy Model-China was separated into the Northern and Southern China versions for this analysis.

**Estimation of salt intake level in 2010**

The Chinese Center for Disease Control and Prevention (China CDC) surveyed dietary salt intake in 2010, but did not report estimates for specific age, sex, or region groups. Therefore, dietary salt intake levels estimated in 2002 by the China CDC Survey are the most recent available nationally-representative data that reported estimates for the subgroups we intended to study (Appendix Table 3). The 2002 China CDC Survey covered 31 provinces, autonomous regions, and municipalities throughout China. A total of 795 residential committees or villages and 68 828 families were sampled, and 192 500 participants were enrolled [[23](#_ENREF_23)]. The salt intake level in 2002 was adjusted 0.06g per year lower for all age, sex, and region groups in order to fit into the "envelope" of the national average level surveyed in 2010 (http://www.chinacdc.cn/mtdx/mxfcrxjbxx/201308/t20130823_87005.htm).

**Appendix Table 3 The age-, gender- and region-specific salt intake level in 2002 and 2010**

|  | **Male (years)** | | | | | **Female (years)** | | | | |
| --- | --- | --- | --- | --- | --- | --- | --- | --- | --- | --- |
|  | **35-44** | **45-54** | **55-64** | **65-74** | **75-94** | **35-44** | **45-54** | **55-64** | **65-74** | **75-94** |
| **Salt intake level in 2002 (g)*** | | | | |  |  |  |  |  |  |
| North | 14.5 | 15.0 | 14.4 | 13.0 | 12.2 | 12.6 | 12.8 | 12.1 | 10.8 | 10.4 |
| South | 12.7 | 13.2 | 12.6 | 11.4 | 10.8 | 11.1 | 11.2 | 10.6 | 9.5 | 9.1 |
| **Salt intake level in 2010 used in the simulation (g)** † | | | | | | | | |  |  |
| North | 14.0 | 14.5 | 13.9 | 12.5 | 11.7 | 12.2 | 12.7 | 12.1 | 10.9 | 10.2 |
| South | 12.2 | 12.7 | 12.1 | 10.9 | 10.2 | 10.6 | 10.7 | 10.1 | 9.0 | 8.6 |

*, Age-, gender- and region-specific salt intake level was estimated by age- and gender-specific salt intake level, region-specific salt intake level, and population number in each region. 1g salt (sodium chloride) = 0.393g sodium;

†, Age-, gender- and region-specific salt intake in 2010 was estimated by subtracting 0.06g per year from the mean salt intake in 2002.

The gold standard method for estimating the dietary sodium intake is a 24-hour urinary test. Both 2002 and 2010 China CDC Surveys estimated daily dietary salt intake using an alternated method of 24-hour recall coupled with a direct cooking salt weighing method. Individuals were asked to provide estimates of the types and amounts of food and drink they had consumed during the previous three 24-hour periods. Then, three-days of cooking salt and cooking salt additives were weighed in each family and the total consumption allocated to individual family members by weighting to individual consumption levels using age- and sex-specific reference equations. The proportion of sodium in each kind of food and condiment was estimated from Chinese food composition tables. In order to assess the validity of this method, we evaluated data from two studies to compare salt intake level estimated by the recall and weighing method and intake level estimated by 24-urine collection to a common method used in both studies--a food table measurement of salt consumption (Appendix Table 4) [[24](#_ENREF_24), [25](#_ENREF_25)]. The ratios of the recall and weighing method and urine collection method to the common comparator were fairly close. This result reassured us that the China CDC Survey dietary recall and weighing method is a reasonable proxy for gold standard measurement of dietary salt consumption in China.

**Appendix Table 4. Comparison of sodium chloride intake level estimated by different methods**

| **Published journal** | **Sample size**  **(n)** | | **Salt intake level (g/day)** | | | | **Ratio compare**  **to food table** | |  |
| --- | --- | --- | --- | --- | --- | --- | --- | --- | --- |
|  |  |  | **Food table** | | **24h urine test** | **24 hours recall modified by weighing method** |  |  |  |
| Journal of the American Dietetic Association (2010) [[24](#_ENREF_24)] |  | 839 |  | 10.1 | 13.3 | -- |  | 1.312 | |
| Chinese Journal of Preventive Medicine (2006) [[25](#_ENREF_25)] |  | 23198 |  | 9.8 | -- | 12.7 |  | 1.296 | |

The population proportion (Appendix Table 5) affected by each policy and the amount of salt lowering were estimated by comparing with the baseline mean and standard deviation of a normal distribution of salt consumption (Appendix Fig 3).

**Appendix Table 5. Estimated mean and proportion of each salt intake level within age-group, by sex and region in 2010**

| **Salt intake**  **Level*** | **Male (years)** | | | | | **Female (years)** | | | | |
| --- | --- | --- | --- | --- | --- | --- | --- | --- | --- | --- |
|  | **35-44** | **45-54** | **55-64** | **65-74** | **75-94** | **35-44** | **45-54** | **55-64** | **65-74** | **75-94** |
| **North** |  |  |  |  |  |  |  |  |  |  |
| **<5 g** | 3.81  (5.7%) | 3.87  (7.7%) | 3.81  (5.7%) | 3.86  (7.4%) | 3.89  (8.7%) | 3.87  (8%) | 3.87  (7.7%) | 3.89  (8.9%) | 3.94 (12.2%) | 3.95 (13.6%) |
| **5 - 5.99 g** | 5.58  (3.7%) | 5.58  (5.2%) | 5.58  (3.7%) | 5.58  (5.0%) | 5.58  (6.0%) | 5.58 (5.4%) | 5.58  (5.2%) | 5.58  (6.2%) | 5.58  (8.7%) | 5.58  (9.7%) |
| **6 - 7.49 g** | 7.05  (8.1%) | 7.05 (11.6%) | 7.05  (8.1%) | 7.05 (11.1%) | 7.05 (13.4%) | 7.05 (12.2%) | 7.05 (11.6%) | 7.05 (13.7%) | 7.04 (19.1%) | 7.04 (21.2%) |
| **7.5 - 8.99 g** | 8.56  (8%) | 8.55 (11.2%) | 8.56  (8%) | 8.55 (10.8%) | 8.55 (12.8%) | 8.55 (11.7%) | 8.55 (11.2%) | 8.55 (13.1%) | 8.54 (17.4%) | 8.54  (19.0%) |
| **>= 9 g** | 16.04 (74.5%) | 14.64 (64.3%) | 16.04 (74.5%) | 14.81 (65.8%) | 14.14 (59.1%) | 14.47 (62.7%) | 14.64 (64.3%) | 14.06 (58.1%) | 13.06 (42.6%) | 12.77 (36.5%) |
| **South** |  |  |  |  |  |  |  |  |  |  |
| **<5 g** | 3.87  (7.8%) | 3.85  (7.1%) | 3.87  (8.0%) | 3.92 (10.5%) | 3.94 (12.5%) | 3.93 (11.3%) | 3.92  (11.0%) | 3.94 (12.8%) | 3.98 (17.5%) | 3.99 (19.9%) |
| **5 - 5.99 g** | 5.58  (5.3%) | 5.58  (4.7%) | 5.58  (5.4%) | 5.58  (7.4%) | 5.58  (8.9%) | 5.58  (8.0%) | 5.58  (7.8%) | 5.58  (9.2%) | 5.58 (12.7%) | 5.58 (14.4%) |
| **6 - 7.49 g** | 7.05 (11.9%) | 7.05 (10.6%) | 7.05 (12.2%) | 7.05 (16.4%) | 7.04 (19.6%) | 7.04 (17.7%) | 7.05 (17.2%) | 7.04 (20.1%) | 7.04  (27%) | 7.03 (30.1%) |
| **7.5 - 8.99 g** | 8.55 (11.5%) | 8.55 (10.3%) | 8.55 (11.7%) | 8.55 (15.3%) | 8.54 (17.8%) | 8.54 (16.3%) | 8.54  (16%) | 8.54 (18.2%) | 8.52 (22.6%) | 8.51 (24.3%) |
| **>= 9 g** | 14.55 (63.5%) | 14.98 (67.3%) | 14.47 (62.7%) | 13.51 (50.5%) | 12.99 (41.2%) | 13.28 (46.7%) | 13.35 (48%) | 12.91 (39.7%) | 12.17 (20.1%) | 11.92 (11.4%) |

*, Sodium chloride intake level

**Appendix Fig 3. Distribution of salt intake level by age-group, sex and regions (estimated by using published means and standard deviations)**

| 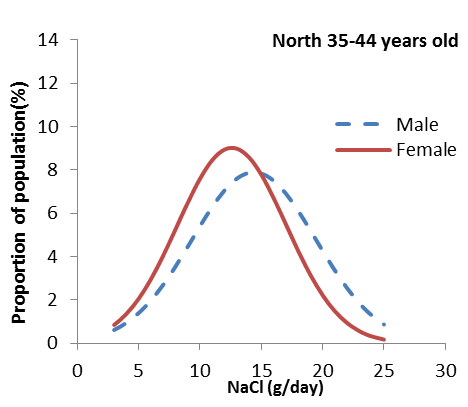 | 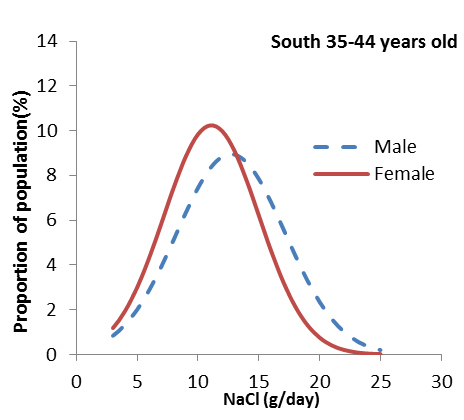 |
| --- | --- |
| 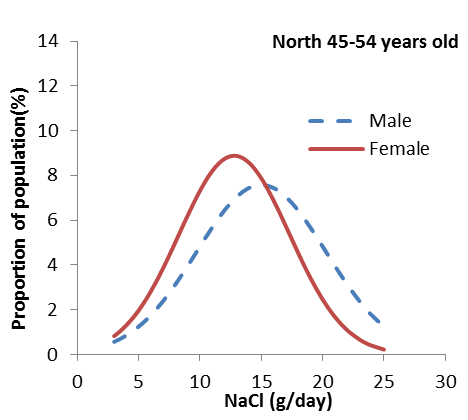 | 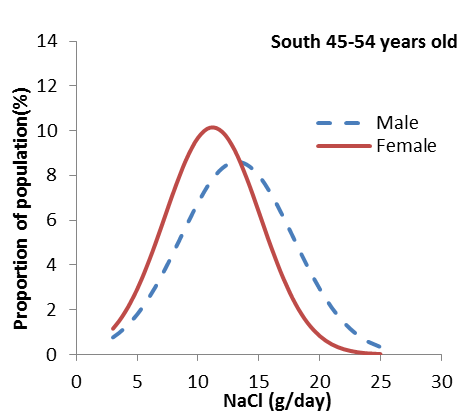 |
| 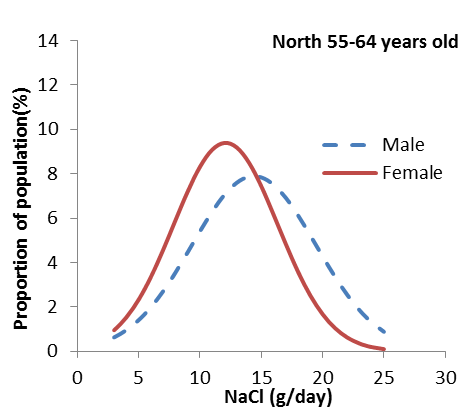 | 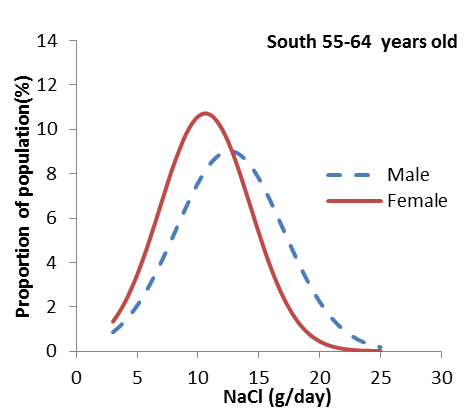 |
| 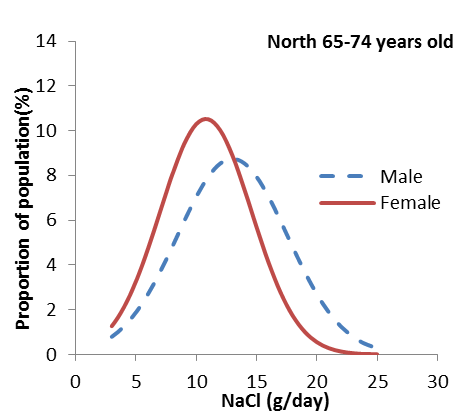 | 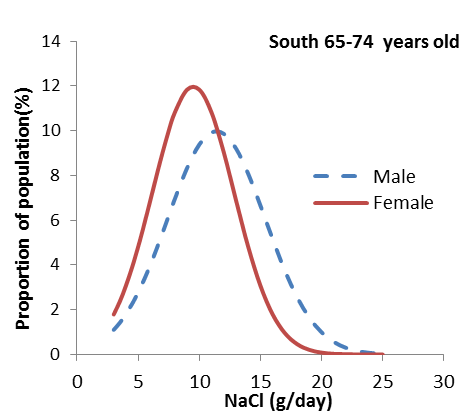 |
| 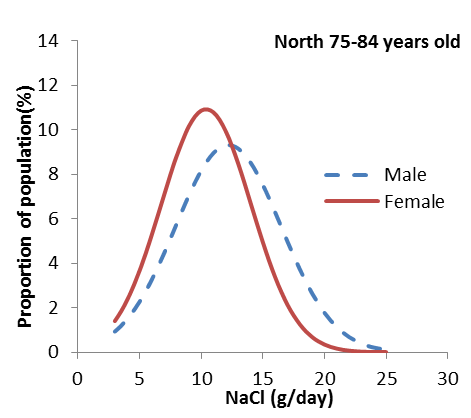 | 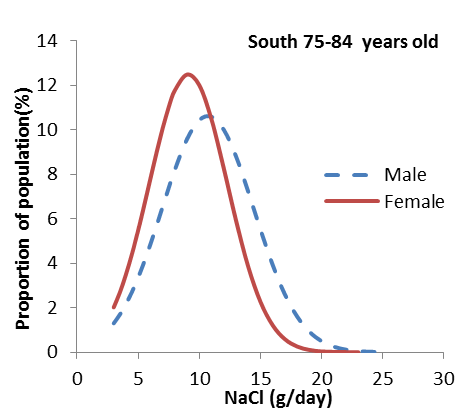 |

NaCl, sodium chloride

**References**

1. Moran A, Zhao D, Gu D, Coxson P, Chen CS, Cheng J, et al. The future impact of population growth and aging on coronary heart disease in China: projections from the Coronary Heart Disease Policy Model-China. BMC Public Health. 2008;8:394. Epub 2008/11/28. doi: 10.1186/1471-2458-8-394.PMID: 19036167.

2. Wang M, Moran AE, Liu J, Coxson PG, Heidenreich PA, Gu D, et al. Cost-effectiveness of optimal use of acute myocardial infarction treatments and impact on coronary heart disease mortality in China. Circ Cardiovasc Qual Outcomes. 2014;7(1):78-85. doi: 10.1161/CIRCOUTCOMES.113.000674.PMID: 24425706.

3. He J, Neal B, Gu D, Suriyawongpaisal P, Xin X, Reynolds R, et al. International collaborative study of cardiovascular disease in Asia: design, rationale, and preliminary results. Ethn Dis. 2004;14(2):260-8. Epub 2004/05/11.PMID: 15132212.

4. Zhao D, Liu J, Wang W, Zeng Z, Cheng J, Liu J, et al. Epidemiological transition of stroke in China: twenty-one-year observational study from the Sino-MONICA-Beijing Project. Stroke. 2008;39(6):1668-74.PMID: 18309149.

5. Gu D, Kelly TN, Wu X, Chen J, Duan X, Huang JF, et al. Blood pressure and risk of cardiovascular disease in Chinese men and women. Am J Hypertens. 2008;21(3):265-72.PMID: 18188156.

6. He J, Gu D, Wu X, Reynolds K, Duan X, Yao C, et al. Major causes of death among men and women in China. N Engl J Med. 2005;353(11):1124-34. doi: 10.1056/NEJMsa050467.PMID: 16162883.

7. Finucane MM, Stevens GA, Cowan MJ, Danaei G, Lin JK, Paciorek CJ, et al. National, regional, and global trends in body-mass index since 1980: systematic analysis of health examination surveys and epidemiological studies with 960 country-years and 9.1 million participants. Lancet. 2011;377(9765):557-67. doi: 10.1016/S0140-6736(10)62037-5.PMID: 21295846.

8. Farzadfar F, Finucane MM, Danaei G, Pelizzari PM, Cowan MJ, Paciorek CJ, et al. National, regional, and global trends in serum total cholesterol since 1980: systematic analysis of health examination surveys and epidemiological studies with 321 country-years and 3.0 million participants. Lancet. 2011;377(9765):578-86. doi: 10.1016/S0140-6736(10)62038-7.PMID: 21295847.

9. Danaei G, Finucane MM, Lu Y, Singh GM, Cowan MJ, Paciorek CJ, et al. National, regional, and global trends in fasting plasma glucose and diabetes prevalence since 1980: systematic analysis of health examination surveys and epidemiological studies with 370 country-years and 2.7 million participants. Lancet. 2011;378(9785):31-40. doi: 10.1016/S0140-6736(11)60679-X.PMID: 21705069.

10. Danaei G, Finucane MM, Lin JK, Singh GM, Paciorek CJ, Cowan MJ, et al. National, regional, and global trends in systolic blood pressure since 1980: systematic analysis of health examination surveys and epidemiological studies with 786 country-years and 5.4 million participants. Lancet. 2011;377(9765):568-77. doi: 10.1016/S0140-6736(10)62036-3.PMID: 21295844.

11. Liu J, Hong Y, D'Agostino RB, Sr., Wu Z, Wang W, Sun J, et al. Predictive value for the Chinese population of the Framingham CHD risk assessment tool compared with the Chinese Multi-Provincial Cohort Study. JAMA. 2004;291(21):2591-9. doi: 10.1001/jama.291.21.2591.PMID: 15173150.

12. Keil U, Kuulasmaa K. WHO MONICA Project: risk factors. Int J Epidemiol. 1989;18(3 Suppl 1):S46-55.PMID: 2807707.

13. Behar S, Tanne D, Abinader E, Agmon J, Barzilai J, Friedman Y, et al. Cerebrovascular accident complicating acute myocardial infarction: incidence, clinical significance and short- and long-term mortality rates. The SPRINT Study Group. Am J Med. 1991;91(1):45-50. Epub 1991/07/01.PMID: 1858828.

14. Witt BJ, Brown RD, Jr., Jacobsen SJ, Weston SA, Yawn BP, Roger VL. A community-based study of stroke incidence after myocardial infarction. Ann Intern Med. 2005;143(11):785-92. Epub 2005/12/07.PMID: 16330789.

15. Amarenco P, Bogousslavsky J, Callahan A, 3rd, Goldstein LB, Hennerici M, Rudolph AE, et al. High-dose atorvastatin after stroke or transient ischemic attack. N Engl J Med. 2006;355(6):549-59.PMID: 16899775.

16. Touze E, Varenne O, Chatellier G, Peyrard S, Rothwell PM, Mas JL. Risk of myocardial infarction and vascular death after transient ischemic attack and ischemic stroke: a systematic review and meta-analysis. Stroke. 2005;36(12):2748-55. Epub 2005/10/29. doi: 10.1161/01.str.0000190118.02275.33.PMID: 16254218.

17. Prosser J, MacGregor L, Lees KR, Diener HC, Hacke W, Davis S. Predictors of early cardiac morbidity and mortality after ischemic stroke. Stroke. 2007;38(8):2295-302. Epub 2007/06/16. doi: 10.1161/strokeaha.106.471813.PMID: 17569877.

18. Appelros P, Gunnarsson KE, Terent A. Ten-year risk for myocardial infarction in patients with first-ever stroke: a community-based study. Acta Neurol Scand. 2011;124(6):383-9. Epub 2011/03/02. doi: 10.1111/j.1600-0404.2011.01500.x.PMID: 21355858.

19. Lakshminarayan K, Schissel C, Anderson DC, Vazquez G, Jacobs DR, Jr., Ezzeddine M, et al. Five-year rehospitalization outcomes in a cohort of patients with acute ischemic stroke: Medicare linkage study. Stroke. 2011;42(6):1556-62. doi: 10.1161/STROKEAHA.110.605600.PMID: 21474798.

20. Lampe FC, Whincup PH, Wannamethee SG, Shaper AG, Walker M, Ebrahim S. The natural history of prevalent ischaemic heart disease in middle-aged men. Eur Heart J. 2000;21(13):1052-62. Epub 2000/06/14. doi: 10.1053/euhj.1999.1866.PMID: 10843823.

21. Rosengren A, Wilhelmsen L, Hagman M, Wedel H. Natural history of myocardial infarction and angina pectoris in a general population sample of middle-aged men: a 16-year follow-up of the Primary Prevention Study, Goteborg, Sweden. J Intern Med. 1998;244(6):495-505. Epub 1999/01/20.PMID: 9893103.

22. Population Census Office under the State Council and Department of Population and Employment Statistics National Bureau of Statistics. Data of 2010 China's Sixth National Population Census. Beijing: China Statistical Press; 2012 2012/12.

23. Wu Y, Huxley R, Li L, Anna V, Xie G, Yao C, et al. Prevalence, awareness, treatment, and control of hypertension in China: data from the China National Nutrition and Health Survey 2002. Circulation. 2008;118(25):2679-86. doi: 10.1161/CIRCULATIONAHA.108.788166.PMID: 19106390.

24. Anderson CA, Appel LJ, Okuda N, Brown IJ, Chan Q, Zhao L, et al. Dietary sources of sodium in China, Japan, the United Kingdom, and the United States, women and men aged 40 to 59 years: the INTERMAP study. J Am Diet Assoc. 2010;110(5):736-45. doi: 10.1016/j.jada.2010.02.007.PMID: 20430135.

25. Li YP, He YN, Zhai FY, Yang XG, Hu XQ, Zhao WH, et al. [Comparison of assessment of food intakes by using 3 dietary survey methods]. Chin J Prev Med. 2006;40(4):273-80.PMID: 17097008.
