# Supplementary material for: Projected Impact of Salt Restriction on Prevention of Cardiovascular Disease in China: A Modeling Study
Source: PLoS One. 2016 Feb 3;11(2):e0146820. doi: 10.1371/journal.pone.0146820 (PMC4739496; doi:10.1371/journal.pone.0146820)
Supplement: S1 Table — (DOCX) [file pone.0146820.s003.docx]

**S1 Table. Results of one-way sensitivity analyses of the potential effects of salt restriction on CVD prevention in China**

|  | **Number of new CVD cases** | **Prevented  new CVD cases** | **CVD incidence rate (per 100 000)** | **Change in CVD incidence rate (%)** | **Number of CVD deaths** | **Prevented CVD deaths** | **Mortality rate (1/100000)** | **Change in CVD  mortality rate (%)** | **QALYs gained** | **Cost saving (Int$, millions)** |
| --- | --- | --- | --- | --- | --- | --- | --- | --- | --- | --- |
| **Base case (average annual number within 2010 to 2019)** | | | | |  |  |  |  |  |  |
| Overall | 8 194 000 | -- | 1 195 | -- | 2 676 000 | -- | 390 | -- | -- | -- |
| North | 4 654 000 | -- | 1 581 | -- | 1 563 000 | -- | 531 | -- | -- | -- |
| South | 3 540 000 | -- | 905 | -- | 1 113 000 | -- | 284 | -- | -- | -- |
| Lower limit of the 95% CI for relative risk of CVD per mmHg change in SBP | | | | | | |  |  |  |  |
| Overall | 8 223 000 | -- | 1 199 | -- | 2 682 000 | -- | 391 | -- | -- | -- |
| North | 4 678 000 | -- | 1 589 | -- | 1 568 000 | -- | 533 | -- | -- | -- |
| South | 3 545 000 | -- | 906 | -- | 1 114 000 | -- | 285 | -- | -- | -- |
| Upper limit of the 95% CI for relative risk of CVD per mmHg change in SBP | | | | | | |  |  |  |  |
| Overall | 8 153 000 | -- | 1 189 | -- | 2 667 000 | -- | 389 | -- | -- | -- |
| North | 4 622 000 | -- | 1 570 | -- | 1 556 000 | -- | 529 | -- | -- | -- |
| South | 3 531 000 | -- | 902 | -- | 1 111 000 | -- | 284 | -- | -- | -- |
| **Impact of achieving salt restriction goals in the whole adult population** | | | | | | |  |  |  |  |
| **Daily salt intake decreases to 9.0 g/day gradually** | | | |  |  |  |  |  |  |  |
| Overall | 7 997 000 | 197 000 | 1 166 | -2.4 | 2 609 000 | 67 000 | 381 | -2.5 | 303 000 | 1 388 |
| North | 4 513 000 | 141 000 | 1 533 | -3.0 | 1 514 000 | 49 000 | 514 | -3.1 | 226 000 | 1 038 |
| South | 3 484 000 | 56 000 | 890 | -1.6 | 1 095 000 | 18 000 | 280 | -1.6 | 77 000 | 350 |
| Lower limit of the 95% CI for relative risk of CVD per mmHg change in SBP | | | | | | |  |  |  |  |
| Overall | 8 069 000 | 154 000 | 1 177 | -1.9 | 2 630 000 | 52 000 | 384 | -1.9 | 258 000 | 1 008 |
| North | 4 570 000 | 108 000 | 1 552 | -2.3 | 1 530 000 | 38 000 | 520 | -2.4 | 191 000 | 738 |
| South | 3 499 000 | 46 000 | 894 | -1.3 | 1 100 000 | 14 000 | 281 | -1.3 | 67 000 | 270 |
| Upper limit of the 95% CI for relative risk of CVD per mmHg change in SBP | | | | | | |  |  |  |  |
| Overall | 7 917 000 | 236 000 | 1 155 | -2.9 | 2 585 000 | 82 000 | 377 | -3.1 | 347 000 | 1 739 |
| North | 4 453 000 | 169 000 | 1 513 | -3.7 | 1 495 000 | 61 000 | 508 | -3.9 | 260 000 | 1 315 |
| South | 3 464 000 | 67 000 | 885 | -1.9 | 1 090 000 | 21 000 | 279 | -1.9 | 87 000 | 424 |
| Lower limit of the 95% CI for SBP change per gram dietary salt change | | | | | |  |  |  |  |  |
| Overall | 8 034 000 | 160 000 | 1 172 | -2.0 | 2 620 000 | 56 000 | 382 | -2.1 | 250 000 | 1 134 |
| North | 4 543 000 | 111 000 | 1 543 | -2.4 | 1 523 000 | 40 000 | 517 | -2.6 | 181 000 | 824 |
| South | 3 491 000 | 49 000 | 892 | -1.4 | 1 097 000 | 16 000 | 280 | -1.4 | 69 000 | 310 |
| Upper limit of the 95% CI for SBP change per gram dietary salt change | | | | | |  |  |  |  |  |
| Overall | 7 981 000 | 213 000 | 1 164 | -2.6 | 2 604 000 | 72 000 | 380 | -2.7 | 329 000 | 1 502 |
| North | 4 502 000 | 152 000 | 1 529 | -3.3 | 1 510 000 | 53 000 | 513 | -3.4 | 245 000 | 1 124 |
| South | 3 479 000 | 61 000 | 889 | -1.7 | 1 094 000 | 19 000 | 280 | -1.7 | 84 000 | 378 |
| **Daily salt intake decreases to 7.5 g/day gradually in ten years** | | | | |  |  |  |  |  |  |
| Overall | 7 869 000 | 325 000 | 1 148 | -4.0 | 2 562 000 | 114 000 | 374 | -4.3 | 491 000 | 2 247 |
| North | 4 447 000 | 207 000 | 1 511 | -4.4 | 1 489 000 | 74 000 | 506 | -4.7 | 331 000 | 1 523 |
| South | 3 422 000 | 118 000 | 875 | -3.3 | 1 073 000 | 40 000 | 274 | -3.6 | 160 000 | 724 |
| Lower limit of the 95% CI for relative risk of CVD per mmHg change in SBP | | | | | |  |  |  |  |  |
| Overall | 7 969 000 | 225 000 | 1 162 | -3.1 | 2 596 000 | 80 000 | 379 | -3.2 | 412 000 | 1 632 |
| North | 4 520 000 | 134 000 | 1 535 | -3.4 | 1 513 000 | 50 000 | 514 | -3.5 | 276 000 | 1 077 |
| South | 3 449 000 | 91 000 | 882 | -2.7 | 1 083 000 | 30 000 | 277 | -2.8 | 136 000 | 555 |
| Upper limit of the 95% CI for relative risk of CVD per mmHg change in SBP | | | | | | |  |  |  |  |
| Overall | 7 762 000 | 432 000 | 1 132 | -4.8 | 2 527 000 | 149 000 | 369 | -5.2 | 566 000 | 2 819 |
| North | 4 371 000 | 283 000 | 1 485 | -5.4 | 1 463 000 | 100 000 | 497 | -6.0 | 383 000 | 1 934 |
| South | 3 391 000 | 149 000 | 867 | -4.0 | 1 064 000 | 49 000 | 272 | -4.2 | 183 000 | 885 |
| Lower limit of the 95% CI for SBP change per gram dietary salt change | | | | | | |  |  |  |  |
| Overall | 7 931 000 | 263 000 | 1 157 | -3.2 | 2 585 000 | 91 000 | 377 | -3.4 | 399 000 | 1 815 |
| North | 4 490 000 | 164 000 | 1 525 | -3.5 | 1 504 000 | 59 000 | 511 | -3.8 | 265 000 | 1 208 |
| South | 3 441 000 | 99 000 | 879 | -2.8 | 1 081 000 | 32 000 | 276 | -2.9 | 134 000 | 607 |
| Upper limit of the 95% CI for SBP change per gram dietary salt change | | | | |  |  |  |  |  |  |
| Overall | 7 842 000 | 352 000 | 1 144 | -4.3 | 2 553 000 | 123 000 | 372 | -4.6 | 531 000 | 2 431 |
| North | 4 430 000 | 224 000 | 1 505 | -4.8 | 1 482 000 | 81 000 | 503 | -5.2 | 358 000 | 1 648 |
| South | 3 412 000 | 128 000 | 872 | -3.6 | 1 071 000 | 42 000 | 274 | -3.8 | 173 000 | 783 |
| **Daily salt intake decreases to 6.0 g/day gradually in ten years** | | | | |  |  |  |  |  |  |
| Overall | 7 739 000 | 455 000 | 1 129 | -5.6 | 2 517 000 | 159 000 | 367 | -5.9 | 680 000 | 3 103 |
| North | 4 381 000 | 273 000 | 1 488 | -5.9 | 1 465 000 | 98 000 | 498 | -6.3 | 435 000 | 1 998 |
| South | 3 358 000 | 182 000 | 858 | -5.1 | 1 052 000 | 61 000 | 269 | -5.5 | 245 000 | 1 105 |
| Lower limit of the 95% CI for relative risk of CVD per mmHg change in SBP | | | | | | |  |  |  |  |
| Overall | 7 867 000 | 356 000 | 1 147 | -4.3 | 2 561 000 | 121 000 | 374 | -4.5 | 569 000 | 2 257 |
| North | 4 469 000 | 209 000 | 1 518 | -4.5 | 1 495 000 | 73 000 | 508 | -4.7 | 361 000 | 1 411 |
| South | 3 398 000 | 147 000 | 869 | -4.1 | 1 066 000 | 48 000 | 272 | -4.3 | 208 000 | 846 |
| Upper limit of the 95% CI for relative risk of CVD per mmHg change in SBP | | | | | | |  |  |  |  |
| Overall | 7 606 000 | 547 000 | 1 109 | -6.7 | 2 471 000 | 196 000 | 360 | -7.3 | 787 000 | 3 889 |
| North | 4 290 000 | 332 000 | 1 457 | -7.2 | 1 433 000 | 123 000 | 487 | -7.9 | 506 000 | 2 537 |
| South | 3 316 000 | 215 000 | 848 | -6.1 | 1 038 000 | 73 000 | 265 | -6.6 | 281 000 | 1 352 |
| Lower limit of the 95% CI for SBP change per gram dietary salt change | | | | | |  |  |  |  |  |
| Overall | 7 829 000 | 365 000 | 1 142 | -4.5 | 2 548 000 | 128 000 | 372 | -4.8 | 549 000 | 2 495 |
| North | 4 438 000 | 216 000 | 1 508 | -4.6 | 1 485 000 | 78 000 | 504 | -5.0 | 347 000 | 1 585 |
| South | 3 391 000 | 149 000 | 867 | -4.2 | 1 063 000 | 50 000 | 272 | -4.5 | 202 000 | 910 |
| Upper limit of the 95% CI for SBP change per gram dietary salt change | | | | | |  |  |  |  |  |
| Overall | 7 701 000 | 493 000 | 1 123 | -6.0 | 2 504 000 | 172 000 | 365 | -6.4 | 736 000 | 3 356 |
| North | 4 358 000 | 296 000 | 1 480 | -6.4 | 1 457 000 | 106 000 | 495 | -6.8 | 471 000 | 2 161 |
| South | 3 343 000 | 197 000 | 854 | -5.6 | 1 047 000 | 66 000 | 268 | -5.9 | 265 000 | 1 195 |
| **Specific salt restriction strategies** | | |  |  |  |  |  |  |  |  |
| **Promoting the use of salt-restriction spoon in the whole population** | | | | | |  |  |  |  |  |
| Overall | 8 011 000 | 183 000 | 1 168 | -2.2 | 2 610 000 | 66 000 | 381 | -2.5 | 401 000 | 1 406 |
| North | 4 561 000 | 93 000 | 1 549 | -2.0 | 1 528 000 | 35 000 | 519 | -2.2 | 221 000 | 784 |
| South | 3 450 000 | 90 000 | 882 | -2.5 | 1 082 000 | 31 000 | 277 | -2.8 | 180 000 | 622 |
| Promoting the use of salt-restriction spoon in the whole population (population adherence rate=75% of adherence in trials) | | | | | | | | | |  |
| Overall | 8 057 000 | 137 000 | 1 175 | -1.7 | 2 626 000 | 50 000 | 383 | -1.9 | 301 000 | 1 058 |
| North | 4 584 000 | 70 000 | 1 557 | -1.5 | 1 536 000 | 27 000 | 522 | -1.7 | 166 000 | 590 |
| South | 3 473 000 | 67 000 | 888 | -1.9 | 1 090 000 | 23 000 | 279 | -2.1 | 135 000 | 468 |
| Promoting the use of salt-restriction spoon in the whole population (population adherence rate=50% of adherence in trials) | | | | | | | |  |  |  |
| Overall | 8 102 000 | 92 000 | 1 182 | -1.1 | 2 642 000 | 34 000 | 385 | -1.3 | 201 000 | 707 |
| North | 4 607 000 | 47 000 | 1 565 | -1.0 | 1 545 000 | 18 000 | 525 | -1.2 | 111 000 | 394 |
| South | 3 495 000 | 45 000 | 893 | -1.3 | 1 097 000 | 16 000 | 280 | -1.4 | 90 000 | 313 |
| Lower limit of the 95% CI for relative risk of CVD per mmHg change in SBP | | | | | | |  |  |  |  |
| Overall | 8 081 000 | 142 000 | 1 179 | -1.7 | 2 631 000 | 51 000 | 384 | -1.9 | 331 000 | 1 023 |
| North | 4 608 000 | 70 000 | 1 565 | -1.5 | 1 542 000 | 26 000 | 524 | -1.7 | 181 000 | 549 |
| South | 3 473 000 | 72 000 | 888 | -2.0 | 1 089 000 | 25 000 | 278 | -2.2 | 150 000 | 474 |
| Upper limit of the 95% CI for relative risk of CVD per mmHg change in SBP | | | | | | |  |  |  |  |
| Overall | 7 933 000 | 220 000 | 1 157 | -2.7 | 2 584 000 | 83 000 | 377 | -3.1 | 468 000 | 1 772 |
| North | 4 509 000 | 113 000 | 1 532 | -2.4 | 1 511 000 | 45 000 | 513 | -2.9 | 260 000 | 1 006 |
| South | 3 424 000 | 107 000 | 875 | -3.0 | 1 073 000 | 38 000 | 274 | -3.4 | 208 000 | 766 |
| Lower limit of the 95% CI for SBP change per gram dietary salt change | | | | | |  |  |  |  |  |
| Overall | 8 050 000 | 144 000 | 1 174 | -1.8 | 2 623 000 | 53 000 | 383 | -2.0 | 317 000 | 1 108 |
| North | 4 581 000 | 73 000 | 1 556 | -1.6 | 1 535 000 | 28 000 | 521 | -1.8 | 175 000 | 616 |
| South | 3 469 000 | 71 000 | 887 | -2.0 | 1 088 000 | 25 000 | 278 | -2.2 | 142 000 | 492 |
| Upper limit of the 95% CI for SBP change per gram dietary salt change | | | | |  |  |  |  |  |  |
| Overall | 7 996 000 | 198 000 | 1 166 | -2.4 | 2 604 000 | 72 000 | 380 | -2.7 | 435 000 | 1 525 |
| North | 4 554 000 | 100 000 | 1 547 | -2.1 | 1 525 000 | 38 000 | 518 | -2.4 | 240 000 | 851 |
| South | 3 442 000 | 98 000 | 880 | -2.8 | 1 079 000 | 34 000 | 276 | -3.1 | 195 000 | 674 |
| **Promoting the use of salt-restriction spoon in people with hypertension only** | | | | | | | | |  |  |
| Overall | 8 054 000 | 140 000 | 1 175 | -1.7 | 2 625 000 | 51 000 | 383 | -1.9 | 292 000 | 1 061 |
| North | 4 582 000 | 72 000 | 1 556 | -1.5 | 1 535 000 | 28 000 | 521 | -1.8 | 162 000 | 600 |
| South | 3 472 000 | 68 000 | 887 | -1.9 | 1 090 000 | 23 000 | 279 | -2.1 | 130 000 | 461 |
| Promoting the use of salt-restriction spoon in people with hypertension only (population adherence rate=75% of adherence in trials) | | | | | | | | | |  |
| Overall | 8 089 000 | 105 000 | 1 180 | -1.3 | 2 637 000 | 39 000 | 385 | -1.5 | 220 000 | 798 |
| North | 4 600 000 | 54 000 | 1 563 | -1.2 | 1 542 000 | 21 000 | 524 | -1.3 | 122 000 | 451 |
| South | 3 489 000 | 51 000 | 892 | -1.4 | 1 095 000 | 18 000 | 280 | -1.6 | 98 000 | 347 |
| Promoting the use of salt-restriction spoon in people with hypertension only (population adherence rate=50% of adherence in trials) | | | | | | | | |  |  |
| Overall | 8 124 000 | 70 000 | 1 185 | -0.9 | 2 649 000 | 27 000 | 386 | -1.0 | 148 000 | 534 |
| North | 4 618 000 | 36 000 | 1 569 | -0.8 | 1 548 000 | 15 000 | 526 | -1.0 | 82 000 | 302 |
| South | 3 506 000 | 34 000 | 896 | -1.0 | 1 101 000 | 12 000 | 281 | -1.1 | 66 000 | 232 |
| Lower limit of the 95% CI for relative risk of CVD per mmHg change in SBP | | | | | | |  |  |  |  |
| Overall | 8 117 000 | 106 000 | 1 184 | -1.3 | 2 644 000 | 38 000 | 386 | -1.4 | 236 000 | 755 |
| North | 4 625 000 | 53 000 | 1 571 | -1.1 | 1 548 000 | 20 000 | 526 | -1.3 | 129 000 | 411 |
| South | 3 492 000 | 53 000 | 893 | -1.5 | 1 096 000 | 18 000 | 280 | -1.6 | 107 000 | 344 |
| Upper limit of the 95% CI for relative risk of CVD per mmHg change in SBP | | | | | | |  |  |  |  |
| Overall | 7 982 000 | 171 000 | 1 164 | -2.1 | 2 602 000 | 65 000 | 380 | -2.4 | 351 000 | 1 376 |
| North | 4 534 000 | 88 000 | 1 540 | -1.9 | 1 520 000 | 36 000 | 516 | -2.3 | 196 000 | 792 |
| South | 3 448 000 | 83 000 | 881 | -2.4 | 1 082 000 | 29 000 | 277 | -2.6 | 155 000 | 584 |
| Lower limit of the 95% CI for SBP change per gram dietary salt change | | | | | | |  |  |  |  |
| Overall | 8 092 000 | 102 000 | 1 180 | -1.2 | 2 638 000 | 38 000 | 385 | -1.4 | 215 000 | 782 |
| North | 4 602 000 | 52 000 | 1 563 | -1.1 | 1 543 000 | 20 000 | 524 | -1.3 | 119 000 | 442 |
| South | 3 490 000 | 50 000 | 892 | -1.4 | 1 095 000 | 18 000 | 280 | -1.6 | 96 000 | 340 |
| Upper limit of the 95% CI for SBP change per gram dietary salt change | | | | |  |  |  |  |  |  |
| Overall | 8 041 000 | 153 000 | 1 173 | -1.9 | 2 619 000 | 57 000 | 382 | -2.1 | 321 000 | 1 161 |
| North | 4 576 000 | 78 000 | 1 554 | -1.7 | 1 532 000 | 31 000 | 520 | -2.0 | 178 000 | 656 |
| South | 3 465 000 | 75 000 | 886 | -2.1 | 1 087 000 | 26 000 | 278 | -2.3 | 143 000 | 505 |
| **Promoting the use of substitute salt in the whole population** | | | | |  |  |  |  |  |  |
| Overall | 7 654 000 | 540 000 | 1 116 | -6.6 | 2 480 000 | 196 000 | 362 | -7.3 | 1 185 000 | 4 126 |
| North | 4 379 000 | 275 000 | 1 488 | -5.9 | 1 459 000 | 104 000 | 496 | -6.7 | 656 000 | 2 308 |
| South | 3 275 000 | 265 000 | 837 | -7.5 | 1 021 000 | 92 000 | 261 | -8.3 | 529 000 | 1 818 |
| Promoting the use of substitute salt in the whole population (population adherence rate=75% of adherence in trials) | | | | | | | |  |  |  |
| Overall | 7 785 000 | 409 000 | 1 135 | -5.0 | 2 527 000 | 149 000 | 369 | -5.6 | 895 000 | 3 126 |
| North | 4 446 000 | 208 000 | 1 510 | -4.5 | 1 484 000 | 79 000 | 504 | -5.1 | 495 000 | 1 747 |
| South | 3 339 000 | 201 000 | 853 | -5.7 | 1 043 000 | 70 000 | 267 | -6.3 | 400 000 | 1 379 |
| Promoting the use of substitute salt in the whole population (population adherence rate=50% of adherence in trials) | | | | | | | |  |  |  |
| Overall | 7 920 000 | 274 000 | 1 155 | -3.3 | 2 576 000 | 100 000 | 376 | -3.7 | 601 000 | 2 105 |
| North | 4 514 000 | 140 000 | 1 533 | -3.0 | 1 510 000 | 53 000 | 513 | -3.4 | 332 000 | 1 175 |
| South | 3 406 000 | 134 000 | 871 | -3.8 | 1 066 000 | 47 000 | 272 | -4.2 | 269 000 | 930 |
| Lower limit of the 95% CI for relative risk of CVD per mmHg change in SBP | | | | | | |  |  |  |  |
| Overall | 7 801 000 | 422 000 | 1 138 | -5.1 | 2 536 000 | 146 000 | 370 | -5.4 | 980 000 | 3 012 |
| North | 4 469 000 | 209 000 | 1 518 | -4.5 | 1 493 000 | 75 000 | 507 | -4.8 | 536 000 | 1 621 |
| South | 3 332 000 | 213 000 | 852 | -6.0 | 1 043 000 | 71 000 | 267 | -6.4 | 444 000 | 1 391 |
| Upper limit of the 95% CI for relative risk of CVD per mmHg change in SBP | | | | | | |  |  |  |  |
| Overall | 7 501 000 | 652 000 | 1 094 | -8.0 | 2 423 000 | 244 000 | 353 | -9.1 | 1 380 000 | 5 177 |
| North | 4 284 000 | 338 000 | 1 455 | -7.3 | 1 424 000 | 132 000 | 484 | -8.5 | 769 000 | 2 950 |
| South | 3 217 000 | 314 000 | 822 | -8.9 | 999 000 | 112 000 | 255 | -10.1 | 611 000 | 2 227 |
| Lower limit of the 95% CI for SBP change per gram dietary salt change | | | | |  |  |  |  |  |  |
| Overall | 8 112 000 | 82 000 | 1 183 | -1.0 | 2 647 000 | 29 000 | 386 | -1.1 | 149 000 | 592 |
| North | 4 611 000 | 43 000 | 1 566 | -0.9 | 1 546 000 | 17 000 | 525 | -1.1 | 84 000 | 348 |
| South | 3 501 000 | 39 000 | 895 | -1.1 | 1 101 000 | 12 000 | 281 | -1.1 | 65 000 | 244 |
| Upper limit of the 95% CI for SBP change per gram dietary salt change | | | | |  |  |  |  |  |  |
| Overall | 7 225 000 | 969 000 | 1 054 | -11.8 | 2 329 000 | 347 000 | 340 | -13.0 | 2 153 000 | 7 359 |
| North | 4 159 000 | 495 000 | 1 413 | -10.6 | 1 379 000 | 184 000 | 468 | -11.8 | 1 193 000 | 4 113 |
| South | 3 066 000 | 474 000 | 784 | -13.4 | 950 000 | 163 000 | 243 | -14.6 | 960 000 | 3 246 |
| **Promoting the use of substitute salt in people with hypertension only** | | | | |  |  |  |  |  |  |
| Overall | 7 776 000 | 418 000 | 1 134 | -5.1 | 2 524 000 | 152 000 | 368 | -5.7 | 876 000 | 3 149 |
| North | 4 439 000 | 215 000 | 1 508 | -4.6 | 1 481 000 | 82 000 | 503 | -5.2 | 487 000 | 1 785 |
| South | 3 337 000 | 203 000 | 853 | -5.7 | 1 043 000 | 70 000 | 267 | -6.3 | 389 000 | 1 364 |
| Promoting the use of substitute salt in people with hypertension only (population adherence rate=75% of adherence in trials) | | | | | | | | |  |  |
| Overall | 7 878 000 | 316 000 | 1 149 | -3.9 | 2 561 000 | 115 000 | 374 | -4.3 | 663 000 | 2 388 |
| North | 4 492 000 | 162 000 | 1 526 | -3.5 | 1 501 000 | 62 000 | 510 | -4.0 | 368 000 | 1 352 |
| South | 3 386 000 | 154 000 | 865 | -4.4 | 1 060 000 | 53 000 | 271 | -4.8 | 295 000 | 1 036 |
| Promoting the use of substitute salt in people with hypertension only (population adherence rate=50% of adherence in trials) | | | | | | | |  |  |  |
| Overall | 7 981 000 | 213 000 | 1 164 | -2.6 | 2 598 000 | 78 000 | 379 | -2.9 | 445 000 | 1 610 |
| North | 4 545 000 | 109 000 | 1 544 | -2.3 | 1 521 000 | 42 000 | 517 | -2.7 | 247 000 | 911 |
| South | 3 436 000 | 104 000 | 878 | -2.9 | 1 077 000 | 36 000 | 275 | -3.2 | 198 000 | 699 |
| Lower limit of the 95% CI for relative risk of CVD per mmHg change in SBP | | | | | | |  |  |  |  |
| Overall | 7 901 000 | 322 000 | 1 152 | -3.9 | 2 572 000 | 110 000 | 375 | -4.1 | 708 000 | 2 253 |
| North | 4 517 000 | 161 000 | 1 534 | -3.4 | 1 511 000 | 57 000 | 513 | -3.6 | 388 000 | 1 230 |
| South | 3 384 000 | 161 000 | 865 | -4.5 | 1 061 000 | 53 000 | 271 | -4.8 | 320 000 | 1 023 |
| Upper limit of the 95% CI for relative risk of CVD per mmHg change in SBP | | | | | |  |  |  |  |  |
| Overall | 7 638 000 | 515 000 | 1 114 | -6.3 | 2 474 000 | 193 000 | 361 | -7.2 | 1 048 000 | 4 054 |
| North | 4 354 000 | 268 000 | 1 479 | -5.8 | 1 450 000 | 106 000 | 493 | -6.8 | 587 000 | 2 340 |
| South | 3 284 000 | 247 000 | 839 | -7.0 | 1 024 000 | 87 000 | 262 | -7.8 | 461 000 | 1 714 |
| Lower limit of the 95% CI for SBP change per gram dietary salt change | | | | | |  |  |  |  |  |
| Overall | 8 061 000 | 133 000 | 1 176 | -1.6 | 2 627 000 | 49 000 | 383 | -1.8 | 278 000 | 1 005 |
| North | 4 586 000 | 68 000 | 1 558 | -1.5 | 1 536 000 | 27 000 | 522 | -1.7 | 154 000 | 568 |
| South | 3 475 000 | 65 000 | 888 | -1.8 | 1 091 000 | 22 000 | 279 | -2.0 | 124 000 | 437 |
| Upper limit of the 95% CI for SBP change per gram dietary salt change | | | | |  |  |  |  |  |  |
| Overall | 7 511 000 | 683 000 | 1 095 | -8.3 | 2 433 000 | 243 000 | 355 | -9.1 | 1 429 000 | 5 092 |
| North | 4 301 000 | 353 000 | 1 461 | -7.6 | 1 432 000 | 131 000 | 486 | -8.4 | 796 000 | 2 893 |
| South | 3 210 000 | 330 000 | 820 | -9.3 | 1 001 000 | 112 000 | 256 | -10.1 | 633 000 | 2 199 |

CVD, cardiovascular disease; CI, confidence interval; Int$, international dollars (Int$1.00 = 3.53 Chinese yuan); QALYs, quality-adjusted life years; SBP, systolic blood pressure.
